# Exceptional-point-encirclement emulation tailoring: multidimensional asymmetric switching of all-fiber devices

**DOI:** 10.1038/s41377-025-02144-x

**Published:** 2026-01-01

**Authors:** Kang Li, Yuchen Zhang, Siwei Wang, Jian Wang

**Affiliations:** 1 Wuhan National Laboratory for Optoelectronics and School of Optical and Electronic Information, Huazhong University of Science and Technology, Wuhan, Hubei China; 2 Hubei Optical Fundamental Research Center, Wuhan, Hubei China; 3 Optics Valley Laboratory, Hubei, Wuhan, Hubei China

**Keywords:** Photonic devices, Fibre optics and optical communications

## Abstract

In non-Hermitian systems, the dynamic encircling of exceptional points (EPs) engenders intriguing chiral phenomena, where the resultant state characteristics are intrinsically dependent upon the encircling handedness. An ingenious approach using simple leaky optical elements has been presented to emulate this chiral behavior without physically encircling an EP. This innovative simplification of EP properties enables a more straightforward implementation of asymmetric switching of polarization and path. Given that photons inherently possess multiple physical degrees of freedom, the research focus has shifted from single-dimensional to multidimensional asymmetric switching. Hence, there is a fundamental challenge of how to achieve multidimensional asymmetric switching through a simple and universally applicable architecture. Here, we propose and experimentally demonstrate a novel topology-optimized architecture, termed EP-encirclement emulation tailoring, enabling multidimensional asymmetric switching. Theoretical analysis reveals that our architecture eliminates the 3-dB inherent loss in conventional architecture by replacing couplers with (de)multiplexers. Building upon this architecture, we harness all-fiber devices to implement a high-performance asymmetric switching of polarization, mode, and orbital angular momentum (OAM). To our knowledge, this is the first experimental demonstration of asymmetric OAM switching to date. Our work provides an efficient topology architecture for emulating dynamic EP encirclement, paving the way for universal and flexible asymmetric switching devices.

## Introduction

Non-Hermitian systems with parity-time (PT) symmetry have attracted widespread attention in various fields of science and engineering^1–6^. This growing interest stems from the longstanding quest to explore and harness the unique phenomena exhibited by such systems, which feature open boundaries or dissipative, nonconservative dynamics^7–10^. By tuning two or more system parameters, the complex eigenvalues of a non-Hermitian Hamiltonian traverse the Riemann surface and coalesce at singularities known as exceptional points (EPs)^11–13^. Notably, EPs are captivating features where multiple eigenvalues and their associated eigenvectors converge, triggering dramatic transitions across these points^14,15^. These transitions result in a range of compelling effects, such as loss-induced transparency^16,17^, single-mode laser, and topological light control^18–21^. Additionally, near exceptional points, the square-root branch point nature of these singularities significantly enhances the sensitivity of various sensing applications^22–28^. Thus, PT symmetry and EPs are not only fundamental physical concepts but also pivotal for advanced applications^29,30^, enabling groundbreaking technologies.

In addition to enhancing sensing capabilities at EPs, the asymmetric switching behavior induced by dynamically encircling EPs represents one of the most captivating topological characteristics of non-Hermitian systems^31–42^. Specifically, in photonics, the dynamical evolution of eigenstates can be realized by gradually altering the geometrical parameters of a dielectric waveguide system^13,43^. This approach has been employed in asymmetric mode switching, where the output mode properties depend exclusively on the encircling direction, independent of the input light states^33,35^. Several innovative strategies for dynamically encircling EPs have been proposed to improve the performance of chiral devices. These include fast encirclement to reduce device size^44^ and Hamiltonian hopping to improve efficiency^33^. Given the inherently multidimensional nature of photons, asymmetric switching of different dimensions holds great potential for advancing photonic processing. For instance, asymmetric polarization switching has been demonstrated and applied to a novel approach for chiral polarizer and data formatting^36^. By dynamically encircling EP across different Riemann sheets, asymmetric switching for orbital angular momentum (OAM) modes has been theoretically studied in 3D waveguides^38^. Despite the progress in achieving asymmetric switching of various dimensions, designing a universal solution that encompasses multiple dimensions remains a substantial challenge.

Moreover, most of the existing approaches depend on encircling EPs, which typically involve complex dynamic encirclement processes. To further simplify the encircling evolution, distilled configurations with simple leaky optical elements have been proposed to emulate the chiral characteristics associated with the EP encirclement, known as “encirclement emulators”^45–47^. Although this concept has achieved asymmetric switching in path and polarization, it still falls short of addressing the challenge of universal multidimensional asymmetric switching mentioned earlier. The topological architecture of the “encirclement emulators” suffers from a 3-dB intrinsic loss due to the leaky coupler. In addition, the currently established architectures typically consist of discrete optical elements in a free-space configuration, which pose issues such as limited operational bandwidth, poor flexibility, bulky size, and high investment. These factors pose significant challenges in improving performances and realizing advanced applications. On the contrary, the optical fiber is considered a sterling transmission medium supporting various dimensional data, and it has been widely applied in multi-dimensional multiplexing^48,49^. In particular, there are fiber devices designed to handle the multiple physical dimensions of photons^50^. This means that the asymmetric switching can be potentially achieved by an all-fiber configuration, which could lead to a flexible device with significant applications.

In this paper, we present a topology-optimized architecture of EP-encirclement emulation tailoring (EET), which adopts the (de)multiplexer configuration to substitute the conventional leaky coupler. Through transfer matrix analysis, we substantiate that the optimized EET architecture achieves near-unity efficiency while overcoming the inherent 3-dB loss limitation in previous approaches. Leveraging this innovative architecture, we successfully demonstrate high-performance multidimensional (including polarization, mode, and phase) asymmetric switching using all-fiber components. Furthermore, by employing diverse fiber (de)multiplexers for high-order modes, we achieve efficient asymmetric switching between these modes and the fundamental mode with superior performance. Notably, while prior theoretical studies have explored asymmetric OAM switching, our work represents the first experimental demonstration of asymmetric OAM switching in broad bandwidth.

## Results

### Topologically optimized architecture

**Fig. 1:**
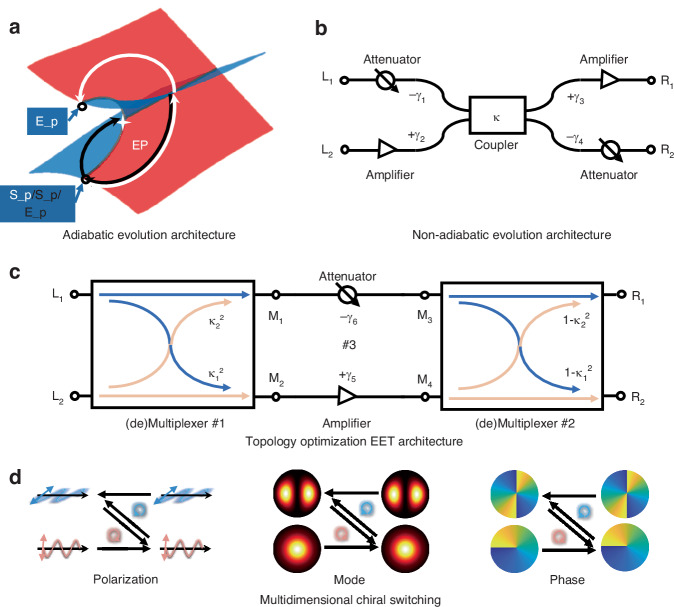
Comparison between conventional adiabatic/non-adiabatic evolution architecture and topology-optimized EET architecture. **a** Conventional adiabatic evolution architecture. This architecture features an exceptional point (EP). **b** Conventional non-adiabatic evolution architecture. This architecture includes two attenuators, two amplifiers and a leaky coupler. **c** Topology-optimized architecture of EP-encirclement emulation tailoring. The system architecture includes an attenuator, an amplifier and two (de)multiplexers. **d** Multi-dimensional (polarization, mode, and phase) asymmetric switching. S_p: starting point; E_p: ending point

To begin, let us understand the fundamental physical principles of asymmetric switching induced by dynamically encircling EP. In a double-coupled waveguide system designed for enabling the asymmetric switching of two orthogonal modes, the characteristic matrix (Hamiltonian *H*) derived from coupled-mode theory forms the foundation of the entire PT-symmetric field in this system^51^:

$$H=\left(\begin{array}{cc}{\beta }_{1}-i{\gamma }_{1} & \kappa \\ \kappa & {\beta }_{2}-i{\gamma }_{2}\end{array}\right)$$

where *β*_1_, *β*_2_, *γ*_1_, *γ*_2_, are the resonant frequencies and relative gain/loss of the two coupled modes. *κ* is the coupling coefficient. The eigenvalues are ${E}_{1,2}={\beta }_{ave}-i{\gamma }_{ave}\pm \sqrt{{({\beta }_{diff}-i{\gamma }_{diff})}^{2}+{\kappa }^{2}}$, where *β*_*ave*_ = (*β*_1_ + *β*_2_)/2, *β*_*diff*_ = (*β*_1_ - *β*_2_)/2, *γ*_*ave*_ = (*γ*_1_ + *γ*_2_)/2, *γ*_*diff*_ = (*γ*_1_ - *γ*_2_)/2. When *β*_*diff*_ = 0 and *γ*_*diff*_ = *κ*, the two eigenvalues degenerate into *E = β*_*ave*_ - *i γ*_*ave*_. As shown in Fig. 1a, when the evolution path encircles the EP, the same starting point reaches different ending points depending on the rotation direction. More detailed theory and methodology can be found in the Supplementary Section S1^43^. Complex encircling path is also mapped onto on-chip waveguide system for verifying the asymmetric switching for mode^35,42^, polarization^32,34^, and OAM^38^. Developing a universal solution that encompasses multiple dimensions remains a significant challenge.

To simplify the evolution processing of encircling EP, researchers have proposed a scheme of emulating EP-encirclement process, which uses leaky linear optical elements to enable the asymmetric switching of path and polarization^45–47^. Figure 1b shows the traditional non-adiabatic evolution architecture, consisting of two attenuators, two amplifiers, and a leaky coupler. The leaky coupler can be simply regarded as a partially directional coupler with coupling coefficient |*κ* | < 1. Consequently, the transmission matrices for forward and backward propagation in this system are as follows:

$$\begin{array}{l}\overrightarrow{T}=\left[\begin{array}{cc}{T}_{\overrightarrow{{L}_{1}-{R}_{1}}} & {T}_{\overrightarrow{{L}_{1}-{R}_{2}}}\\ {T}_{\overrightarrow{{L}_{2}-{R}_{1}}} & {T}_{\overrightarrow{{L}_{2}-{R}_{2}}}\end{array}\right]=\left[\begin{array}{cc}10\ast \,\mathrm{lg}(1-{\kappa }^{2})-{\gamma }_{1}+{\gamma }_{3} & 10\ast \,\mathrm{lg}({\kappa }^{2})-{\gamma }_{1}-{\gamma }_{4}\\ 10\ast \,\mathrm{lg}({\kappa }^{2})+{\gamma }_{2}+{\gamma }_{3} & 10\ast \,\mathrm{lg}(1-{\kappa }^{2})+{\gamma }_{2}-{\gamma }_{4}\end{array}\right]\\ \overleftarrow{T}=\left[\begin{array}{cc}{T}_{\overleftarrow{{L}_{1}-{R}_{1}}} & {T}_{\overleftarrow{{L}_{2}-{R}_{1}}}\\ {T}_{\overleftarrow{{L}_{1}-{R}_{2}}} & {T}_{\overleftarrow{{L}_{2}-{R}_{2}}}\end{array}\right]=\left[\begin{array}{cc}10\ast \,\mathrm{lg}(1-{\kappa }^{2})-{\gamma }_{1}+{\gamma }_{3} & 10\ast \,\mathrm{lg}({\kappa }^{2})+{\gamma }_{2}+{\gamma }_{3}\\ 10\ast \,\mathrm{lg}({\kappa }^{2})-{\gamma }_{1}-{\gamma }_{4} & 10\ast \,\mathrm{lg}(1-{\kappa }^{2})+{\gamma }_{2}-{\gamma }_{4}\end{array}\right]\end{array}$$

where ${T}_{\overrightarrow{{\rm{i}}-{\rm{j}}}}({T}_{\overleftarrow{{\rm{i}}-{\rm{j}}}})$ represents the transmission from i(j) port to j(i) port. Amplifiers are not selected in the experimental setup of the “encircling simulator”, namely: *γ*_2_ = *γ*_3_ = 0 dB. We choose the attenuators with varying degrees *γ*_1_ = 15 dB, *γ*_4_ = 16 dB. The transmission matrices of the system are:

$$\begin{array}{l}\overrightarrow{{T}_{Tra}}=\left[\begin{array}{cc}10\ast \,\mathrm{lg}(1-{\kappa }^{2})-15 & 10\ast \,\mathrm{lg}({\kappa }^{2})-31\\ 10\ast \,\mathrm{lg}({\kappa }^{2}) & 10\ast \,\mathrm{lg}(1-{\kappa }^{2})-16\end{array}\right]\\ \overleftarrow{{T}_{Tra}}=\left[\begin{array}{cc}10\ast \,\mathrm{lg}(1-{\kappa }^{2})-15 & 10\ast \,\mathrm{lg}({\kappa }^{2})\\ 10\ast \,\mathrm{lg}({\kappa }^{2})-31 & 10\ast \,\mathrm{lg}(1-{\kappa }^{2})-16\end{array}\right]\end{array}$$

Additionally, the required coupler provides the system with extensive flexibility, with its performances shown as follows:

$$\overrightarrow{CT}=\left[\begin{array}{l}10\ast \,\mathrm{lg}({\kappa }^{2})-10\ast \,\mathrm{lg}(1-{\kappa }^{2})-16\\ 10\ast \,\mathrm{lg}(1-{\kappa }^{2})-10\ast \,\mathrm{lg}({\kappa }^{2})-16\end{array}\right],\overleftarrow{CT}=\left[\begin{array}{l}10\ast \,\mathrm{lg}(1-{\kappa }^{2})-10\ast \,\mathrm{lg}({\kappa }^{2})-15\\ 10\ast \,\mathrm{lg}({\kappa }^{2})-10\ast \,\mathrm{lg}(1-{\kappa }^{2})-15\end{array}\right]$$

$$\overrightarrow{Loss}=\,\min \{-(10\ast \,{\text{lg}}(1-{\kappa }^{2})-15),-(10\ast \,{\text{lg}}({\kappa }^{2}))\},\overleftarrow{Loss}=\,\min \{-(10\ast \,{\text{lg}}(1-{\kappa }^{2})-16),-(10\ast \,{\text{lg}}({\kappa }^{2}))\}$$

Supplementary Section S2 shows the crosstalk and the minimum transformation loss for couplers with varying coupling efficiency *κ*^2^. It is observed that when the coupling efficiency approaches either 100% or 0, the crosstalk values have opposite signs, indicating the presence of two output modes rather than asymmetric switching. Additionally, it is evident that when the coupling efficiency is around 50%, the mode crosstalk values are roughly balanced, both staying near -*γ*_1_ and -*γ*_4_. The corresponding maximum transmission efficiency, however, is limited to 50%. Therefore, this traditional non-adiabatic evolution architecture requires a trade-off between crosstalk and minimum transformation loss, making it impossible to achieve optimal performance for both simultaneously.

Alternatively, we further propose the topology-optimized EET architecture, as illustrated in Fig. 1c. In this optimized architecture, (de)multiplexers are used in place of coupler to function as leakage devices, and the order of components is rearranged to optimize the light propagation path. This optimization reduces device count and minimizes system loss, all while preserving the topological functionality of the system. The forward and reverse transmission matrices of (de)multiplexer are as follows:

$$\overrightarrow{{T}_{MSC}}=\left[\begin{array}{cc}\overrightarrow{{T}_{{L}_{1}{M}_{1}}} & \overrightarrow{{T}_{{L}_{1}{M}_{2}}}\\ \overrightarrow{{T}_{{L}_{2}{M}_{1}}} & \overrightarrow{{T}_{{L}_{2}{M}_{2}}}\end{array}\right]=\left[\begin{array}{cc}10\ast \,\mathrm{lg}(1-{\kappa }_{1}) & 10\ast \,\mathrm{lg}({\kappa }_{1})\\ 10\ast \,\mathrm{lg}({\kappa }_{2}) & 10\ast \,\mathrm{lg}(1-{\kappa }_{2})\end{array}\right]$$

$$\overleftarrow{{T}_{MSC}}=\left[\begin{array}{cc}{T}_{{\overleftarrow{{L}_{1}M}}_{1}} & {T}_{{\overleftarrow{{L}_{2}M}}_{1}}\\ {T}_{{\overleftarrow{{L}_{1}M}}_{2}} & {T}_{{\overleftarrow{{L}_{2}M}}_{2}}\end{array}\right]=\left[\begin{array}{cc}{T}_{\overrightarrow{{L}_{1}{M}_{1}}} & {T}_{\overrightarrow{{L}_{2}{M}_{1}}}\\ {T}_{\overrightarrow{{L}_{1}{M}_{2}}} & {T}_{\overrightarrow{{L}_{2}{M}_{2}}}\end{array}\right]=\left[\begin{array}{cc}10\ast \,\mathrm{lg}(1-{\kappa }_{1}) & 10\ast \,\mathrm{lg}({\kappa }_{2})\\ 10\ast \,\mathrm{lg}({\kappa }_{1}) & 10\ast \,\mathrm{lg}(1-{\kappa }_{2})\end{array}\right]$$

where *κ*_1_ and *κ*_2_ represent the coupling efficiencies of the (de)multiplexers. Additionally, this architecture only requires an attenuator and an amplifier, reducing the number of components compared to the traditional architecture. Similarly, we evaluate the performance of this architecture through its transmission matrix. The forward transmission matrix is as follows:

$$\overrightarrow{{T}_{Top}}={T}_{\#1\to \#3\to \#2}=\left[\begin{array}{cc}{T}_{\overrightarrow{{L}_{1}{R}_{1}}} & {T}_{\overrightarrow{{L}_{1}{R}_{2}}}\\ {T}_{\overrightarrow{{L}_{2}{R}_{1}}} & {T}_{\overrightarrow{{L}_{2}{R}_{2}}}\end{array}\right]=\left[\begin{array}{cc}{T}_{{\overrightarrow{{L}_{1}{M}_{3}R}}_{1}}+{T}_{{\overrightarrow{{L}_{1}{M}_{4}R}}_{1}} & {T}_{{\overrightarrow{{L}_{1}{M}_{3}R}}_{2}}+{T}_{{\overrightarrow{{L}_{1}{M}_{4}R}}_{2}}\\ {T}_{{\overrightarrow{{L}_{2}{M}_{3}R}}_{1}}+{T}_{{\overrightarrow{{L}_{2}{M}_{4}R}}_{1}} & {T}_{{\overrightarrow{{L}_{2}{M}_{3}R}}_{2}}+{T}_{{\overrightarrow{{L}_{2}{M}_{4}R}}_{2}}\end{array}\right]$$

Due to the huge loss of optical attenuator (*γ*_6_ > 60 dB), the transmission of experiencing optical attenuator can be ignored to further simplify the transmission matrix $\overrightarrow{{T}_{Top}}$:

$$\begin{array}{l}\overrightarrow{{T}_{Top}}={T}_{\#1\to \#3\to \#2}=\left[\begin{array}{cc}{T}_{\overrightarrow{{L}_{1}{R}_{1}}} & {T}_{\overrightarrow{{L}_{1}{R}_{2}}}\\ {T}_{\overrightarrow{{L}_{2}{R}_{1}}} & {T}_{\overrightarrow{{L}_{2}{R}_{2}}}\end{array}\right]\approx \left[\begin{array}{cc}{T}_{{\overrightarrow{{L}_{1}{M}_{4}R}}_{1}} & {T}_{{\overrightarrow{{L}_{1}{M}_{4}R}}_{2}}\\ {T}_{{\overrightarrow{{L}_{2}{M}_{4}R}}_{1}} & {T}_{{\overrightarrow{{L}_{2}{M}_{4}R}}_{2}}\end{array}\right]\\ =\left[\begin{array}{cc}10\ast \,\mathrm{lg}({{\kappa }_{1}}^{2})+10\ast \,\mathrm{lg}(1-{{\kappa }_{2}}^{2})+{\gamma }_{5} & 10\ast \,\mathrm{lg}({{\kappa }_{1}}^{2})+10\ast \,\mathrm{lg}({{\kappa }_{2}}^{2})+{\gamma }_{5}\\ 10\ast \,\mathrm{lg}(1-{{\kappa }_{2}}^{2})+10\ast \,\mathrm{lg}(1-{{\kappa }_{2}}^{2})+{\gamma }_{5} & 10\ast \,\mathrm{lg}(1-{{\kappa }_{2}}^{2})+10\ast \,\mathrm{lg}({{\kappa }_{2}}^{2})+{\gamma }_{5}\end{array}\right]\end{array}$$

Similarly, the amplifier is not used, and we select different (de)multiplexers with common performances, specifically: *γ*_5_ = 0, *κ*_1_^2^ = 0.97, *κ*_2_^2^ = 0.98. The matrix can be further expressed as:

$$\overrightarrow{{T}_{Top}}=\left[\begin{array}{cc}-17.12 & -0.22\\ -33.98 & -17.08\end{array}\right],\,\overleftarrow{{T}_{Top}}=\left[\begin{array}{cc}-17.12 & -32.22\\ -0.22 & -15.36\end{array}\right]$$

The similar equation derivation yields the reverse transmission matrix (more detailed derivation in Supplementary Section S3). ${T}_{\overrightarrow{{L}_{1}{R}_{2}}}\gg {T}_{\overrightarrow{{L}_{1}{R}_{1}}},{T}_{\overrightarrow{{L}_{2}{R}_{2}}}\gg {T}_{\overrightarrow{{L}_{2}{R}_{1}}}$ show that the output primarily occurs at port R_2_ when light forward propagates. ${T}_{\overleftarrow{{L}_{1}{R}_{1}}}\gg {T}_{\overleftarrow{{L}_{2}{R}_{1}}},{T}_{\overleftarrow{{L}_{1}{R}_{2}}}\gg {T}_{{\overrightarrow{{L}_{2}R}}_{2}}$ display that the output primarily occurs at port L_1_ when light backward propagates. This result clearly demonstrates the implementation of asymmetric switching by the proposed architecture. Notably, the minimum transformation loss of the system is just 0.22 dB, corresponding only to the inherent loss of the two couplers. Fig. S3 in Supplementary Section S2 illustrates the relationship between minimum transformation loss and crosstalk as a function of the coupling efficiency of the (de)multiplexer. As the coupling efficiency increases, the minimum transformation loss and the crosstalk simultaneously decrease. This indicates that with high-efficiency (de)multiplexers, the optimized topology can achieve virtually lossless performance while maintaining low crosstalk. Therefore, the proposed architecture eliminates the need to compromise between maximizing transmission efficiency and minimizing crosstalk. Notably, our architecture supports asymmetric switching between any two states, provided that a corresponding multiplexer is properly implemented.

### All-fiber multi-dimensional asymmetric switching

The proposed topology-optimized EET architecture employs two (de)multiplexers and a variable optical attenuator (VOA), which can be implemented using corresponding free-space optical components. However, free-space components typically have limited bandwidth, larger volume and more components to achieve multidimensional (de)multiplexing. Leveraging the flexibility of all-fiber devices, we can readily envision a fully fiber-based version of this setup. By replacing (de)multiplexers of different dimensions, this design enables asymmetric switching across multiple dimensions (such as polarization, mode, and phase), as shown in Fig. 1d. In a sense, this topology-optimized EET architecture demonstrates robustness and wide applicability.

### All-fiber chiral polarizer

**Fig. 2:**
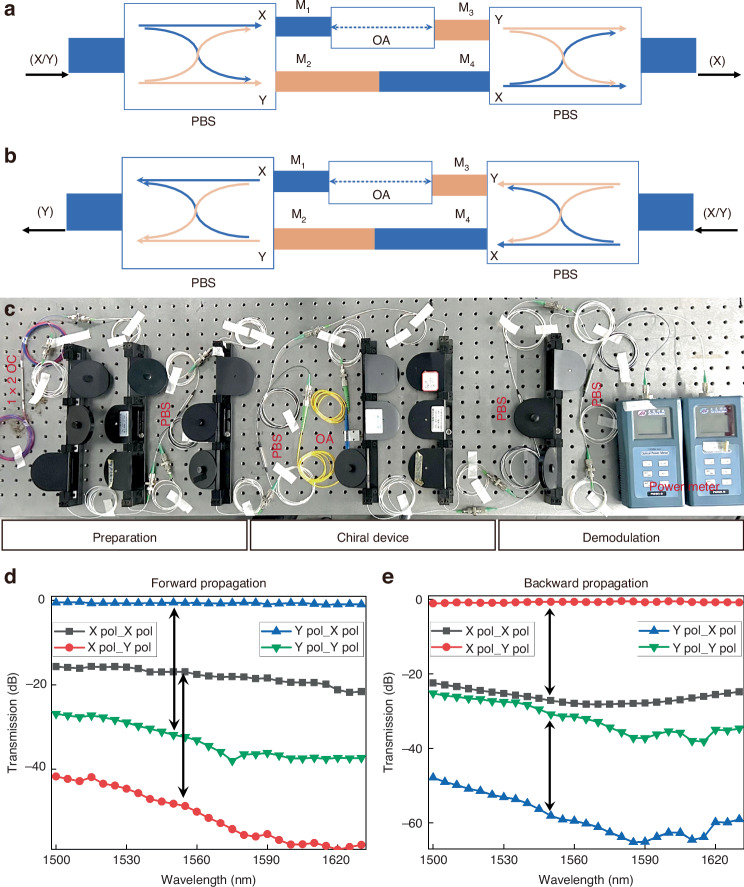
All-fiber chiral polarizer. Proposed scheme realizing chiral polarizer by topology-optimized EET architecture, when lights with dual polarizations propagate **a** forward and **b** backward. **c** Experimental setup for chiral polarizer including dual-polarization preparation, chiral device, and dual-polarization demodulation. **d,** **e** Experimental measurement of **d** forward and **e** backward transmission spectra of chiral polarizer. OA, optical attenuator, OC optical coupler, PBS polarization beam splitter

Based on the topology architecture described above, Fig. 2a, b illustrate our all-fiber chiral polarizer, comprising two commercial polarization beam splitters (PBSs) and a fiber attenuator. The beam combining ports of the two PBSs serve as the input and output ports of the chiral polarizer. The demultiplexer ports are cross-connected, with the X port connected to the Y port. And a fiber attenuator is inserted in one path. When X/Y-polarized light is coupled into the left input port, the normally coupled X-polarized light and any additional Y-polarized light from M_1_ are highly attenuated by the fiber attenuator before reaching M_3_. Conversely, on the other path, the Y-polarized light and additional X-polarized light can pass directly through M_2_ to M_4_ without loss. As a result, the power at M_3_ is negligible compared to M_4_, and the output primarily comes from M_4_, corresponding to the X-polarized component of the PBS. Thus, the output light exiting from the right side is always X-polarized. Using a similar analysis, it can be concluded that when X/Y-polarized light enters from the right, the output from the left port is always Y-polarized. Figure 2c shows the experimental setup of the chiral polarizer, including the X/Y polarization preparation, demodulation, and the all-fiber chiral polarizer. The X/Y polarization preparation and demodulation units use PBSs to generate and demodulate the X/Y-polarized light. Several polarization controllers (PCs) adjust the fiber polarization to ensure correct alignment. A 1×2 splitter divides the light from a tunable laser into two paths, allowing polarization switching without altering the polarization state. By tuning the laser wavelength, two optical power meters continuously measure the output power of each polarization. Figure 2d, e display the measured transmission spectra for forward and backward transmission with different polarization states. The label “m pol_n pol” denotes the transmission from input m polarization to output n polarization. As shown in Fig. 2d, for forward X-polarized input, X pol_X pol $\gg$ X pol_Y pol indicating that the X polarization dominates the output. Across the wavelength range of 1500–1630 nm, this dominance reaches up to 20 dB, showing high purity. For forward Y-polarized input, Y pol_X pol $\gg$ Y pol_Y pol shows that Y polarization transforms into X polarization during transmission, with X polarization dominating the output. Thus, in forward transmission, the polarization is locked to X polarization, while for backward transmission, it locks to Y polarization, as shown in Fig. 2e. Interestingly, transmission with polarization conversion is always higher than without, with the minimum transformation loss being less than 1 dB. Experimental results strongly support the chiral polarization switching and validate the chiral polarization locking. However, as discussed, this topology also faces the issue of uneven output amplitudes, where one input polarization exhibits significant attenuation, reducing the signal-to-noise ratio and thus posing a challenge for practical applications. This limitation can be mitigated by placing an amplifier at position *γ*_5_ to uniformly boost all output power levels, reducing the impact of amplitude imbalance on real-world applications.

### Asymmetric switching for various spatial lights

**Fig. 3:**
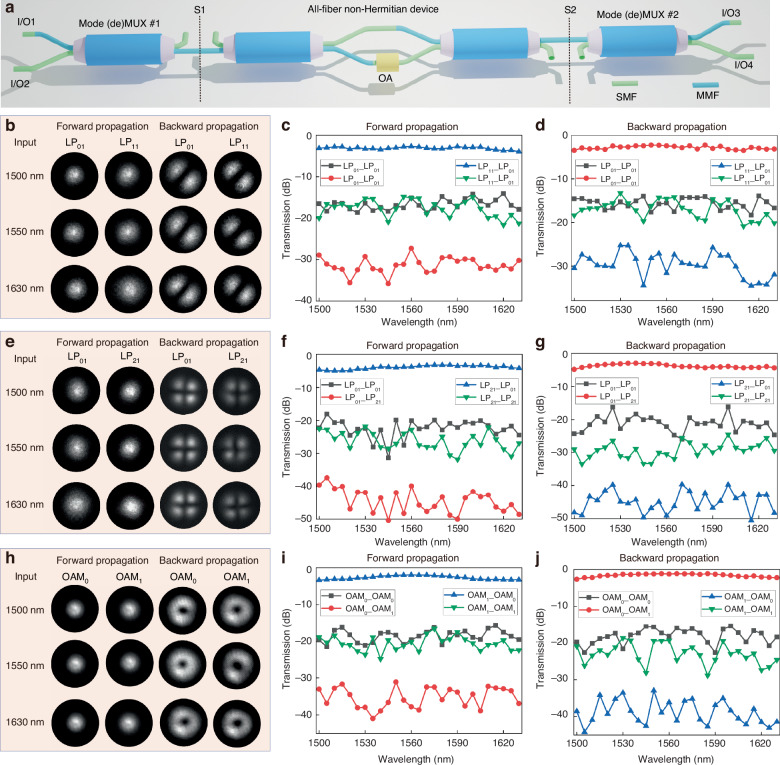
Asymmetric switching for various spatial lights. **a** Proposed topology-optimized EET architecture enabling asymmetric switching for various spatial modes. **b**–**j** Experimental measurement results including **b**, **e**, **h** intensity profiles of output modes, **c**, **f**, **i** forward transmission spectra and **d**, **g**, **j** backward transmission spectra. The spatial modes are **b**–**d** LP_11_ mode, **e**–**g** LP_21_ mode, and **b**–**d** OAM_1_ mode, respectively. In order to avoid overexposure of the output intensity profiles, the output intensity profiles of the second and third column mode fields are measured when the input laser power is reduced by 10 dB, compared to the output intensity profiles of the first and fourth column mode fields. SMF single-mode fiber, MMF multi-mode fiber

The spatial dimension, as an emerging degree of freedom, offers a promising avenue for enhancing the capacity of optical communication systems^52^. Spatial mode conversion is essential for enabling more sophisticated information processing through multi-channel data exchange in communication. While various on-chip systems have been proposed to achieve asymmetric switching between odd and even waveguide modes with single, dual, and triple waveguides, asymmetric switching for spatial modes such as LP and OAM modes and fundamental modes remains unexplored. Our proposed EET architecture can be readily applied to achieve asymmetric switching of various spatial modes. Figure 3a illustrates the topological architecture for spatial asymmetric switching using homemade all-fiber mode-selective couplers (MSCs). The all-fiber MSCs serve as (de)multiplexers, which can generate and demodulate different spatial modes (more details about all-fiber device design and fabrication can be found in Supplementary Section S4). As shown in Fig. 3a, when light enters through ports I/O1 and I/O3, the output modes are the fundamental modes at the locations S1 and S2. When light enters through ports I/O2 and I/O4, the output modes are high-order spatial modes at the locations S1 and S2. Due to its non-negligible crosstalk, the custom all-fiber multiplexer can effectively function as an imperfect (leaky) device within the optimized architecture. The corresponding experimental setup can be found in Supplementary Section S5.

Figure 3b shows the intensity profiles of the output modes for asymmetric switching of LP_01_ and LP_11_ modes at different wavelengths (More details about the measurement setup can be found in Supplementary Section S6). Due to the stronger intensity of the converted mode relative to the unconverted mode, uniform output laser power causes overexposure in the mode profile measurement. To address this, the input laser power for high-intensity output profiles is reduced by 10 dB. The intensity profiles reveal that when light is input forward (from left to right in Fig. 3a), the output mode at S2 is consistently LP_01_. When light is input in the reverse direction (from right to left in Fig. 3a), the output mode at S1 is consistently LP_11_. This clearly demonstrates that the output mode state depends solely on the propagation direction, independent of the input mode. Figure 3c, d shows the forward and backward transmission spectra over the wavelength range of 1500–1630 nm, demonstrating high transmission efficiency and high mode purity. The device achieves a minimum transformation loss of less than 3 dB and mode crosstalk below -11 dB. Compared to devices operating in the polarization switching, the asymmetric mode-switching device exhibits higher transmission loss, primarily due to the greater loss associated with the homemade mode multiplexer, as opposed to the lower-loss commercial polarization multiplexers. Future improvements in mode multiplexer fabrication processes could reduce this loss, enhancing the overall performance of the device. Our architecture can also be adapted for asymmetric switching of higher-order modes by replacing the higher-order mode multiplexer. Figure 3e-g show the intensity profiles, forward transmission spectra, and backward transmission spectra for asymmetric transmission of LP_01_ and LP_21_ modes. The measured results demonstrate that this device outputs the LP_01_ mode at the right port and the LP_21_ mode at the left port. Due to lower crosstalk in the LP_21_ multiplexer compared to the LP_11_ multiplexer, asymmetric transmission between LP_01_ and LP_21_ modes achieves slightly higher mode purity, reaching <-15 dB. Similarly, by replacing the LP mode multiplexer with an OAM multiplexer, the topological architecture can achieve asymmetric switching for OAM_0_ and OAM_1_ modes with favorable performance, as shown in detail in Fig. 3h–j. The subscript of OAM in this work represents the azimuthal order and omits the radial order. The corresponding experimental setup for characterizing the intensity profiles can be found in Supplementary Section S6.

### Asymmetric switching for various phase lights

**Fig. 4:**
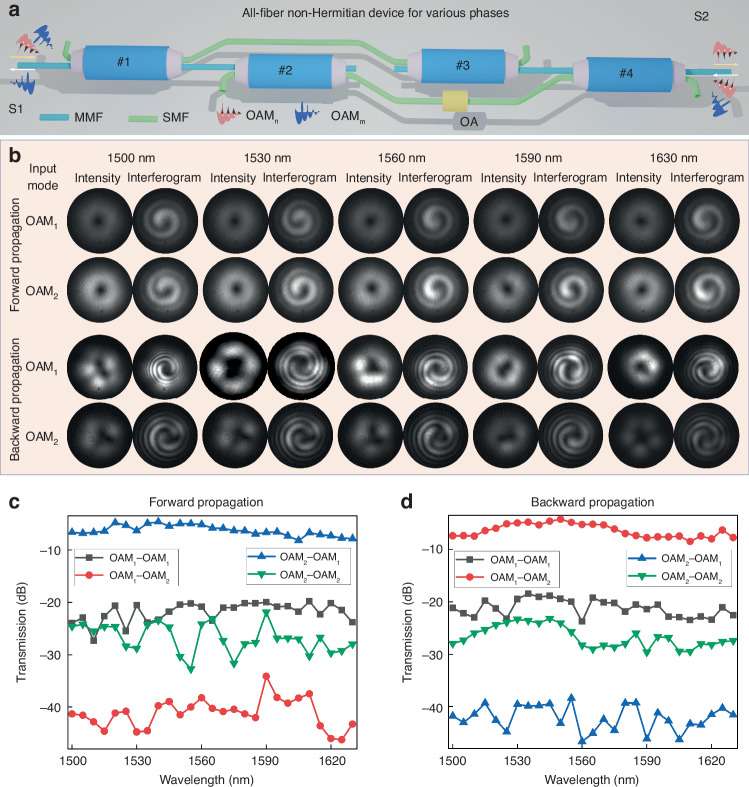
Asymmetric switching for various phase lights. **a** Proposed topology-optimized EET architecture enabling asymmetric switching for lights with various phases. **b**–**d** Experimental measurement results including **b** intensity profiles, interferograms of output modes, **c** forward transmission spectra, and **d** backward transmission spectra. The phase lights are OAM_1_ mode and OAM_2_ mode. In order to prevent overexposure of the output intensity profiles, the output mode profiles of the second and third lines are measured when the input laser power is reduced by 10 dB, compared to the output mode profiles of the first and fourth lines

OAM provides an additional degree of freedom for optical communications, characterized by a spiral phase front exp(-*ilθ*) with theoretically infinite orders. Here, we select OAM modes with different orders to represent light beams with varying phase distributions. Similar to other dimensions, the asymmetric OAM switching is crucial for enabling more complex information processing, such as data formatting. However, the currently established OAM manipulation devices are typically composed of discrete optical elements in free-space configurations, which present challenges like high costs and bulky sizes, hindering their implementation in practical engineering applications and commercial products. Although some integrated solutions, such as femtosecond laser-fabricated 3D chips, have been proposed for asymmetric OAM switching, the complex refractive index modulation makes experimental demonstrations highly intricate. To address this challenge, we propose a flexible solution based on the topology-optimized EP-encirclement emulation scheme implemented in an all-fiber device. This approach enables asymmetric switching between arbitrary OAM modes and offers well scalability. As shown in Fig. 4a, the device consists of four all-fiber mode-selective couplers and one OA. MSC #1 and MSC #4 correspond to the OAM_m_, while MSC #2 and MSC #3 correspond to another OAM_n_ (m > n). When OAM_n_ and OAM_m_ are input from the left side, the majority of the light coupled from the OAM_n_ mode and a small portion of leaked light from the OAM_m_ mode passes through MSC #1 and MSC #2, then propagate via a single-mode fiber and an OA to MSC #4. The OA attenuates the light to a negligible level. Meanwhile, the majority of the light coupled from the OAM_m_ mode and a small fraction of leaked light from the OAM_n_ mode are transmitted to MSC #3 and coupled into the OAM_n_ mode in the multi-mode fiber via a single-mode fiber. The OAM_n_ mode then passes through MSC #4. As a result, the output mode at the right port is OAM_n_. Conversely, when OAM_n_ and OAM_m_ are input from the right side through MSC #4, a small fraction of leaked light from the OAM_n_ mode and the majority of the light coupled from the OAM_m_ mode propagates through the single-mode fiber to the OA, where they are likewise attenuated. The remaining light passes through MSC #3 and MSC #1 and is coupled into the OAM_m_ mode in the output multimode fiber. Therefore, the output at the left port is OAM_m_.

To experimentally validate this concept, we select OAM_1_ and OAM_2_ as the demonstrations. The experimental setup is detailed in Supplementary Section S5. Figure 4b illustrates the output intensity profiles and interferograms at different wavelengths when OAM_1_ and OAM_2_ are input from the left or right port, respectively. Clear asymmetric switching between OAM_1_ and OAM_2_ is observed: the phase of the right-side output locks to OAM_1_, while the left-side output locks to OAM_2_, regardless of the input phase. Figure 4c, d show the transmission spectra of the device from 1500 nm to 1630 nm, further confirming the asymmetric OAM switching. Within this wavelength range, the device exhibits the insertion loss of less than 8 dB and the crosstalk below -11.3 dB. By optimizing the performance of the MSCs, the overall performance of the device can be further improved.

## Discussion

Within the proposed topology-optimized EET architecture, we have already achieved high-performance multidimensional asymmetric switching using various all-fiber devices, notably including asymmetric OAM switching. Notably, since the performance of both the attenuator and the (de)multiplexer components is independent of the input optical power, the proposed device reliably demonstrates high-efficiency asymmetric switching across varying input light intensities (See Supplementary Section S7). If this topological architecture is applied to photonic integrated circuits (PICs), the design could yield breakthrough results in device size reduction (details in Supplementary Section S8). Currently, most PICs employ adiabatic, gradual encircling EP schemes to achieve asymmetric mode-switching performance improvements, including encircling moving-EP^35^ and fast encirclement^44^, with a reported minimum size of 57 μm. In contrast, implementing our optimized topological architecture on an integrated photonic platform, along with previously reported compact mode multiplexers and attenuators^53,54^, could enable high-performance, compact non-Hermitian devices. This non-Hermitian device is anticipated to realize on-chip asymmetric mode transmission within a compact footprint of approximately 14.25 μm, while maintaining both high transmission efficiency and mode purity. It represents a significant advancement in the development of compact and efficient on-chip non-Hermitian photonic systems. Moreover, with the assistance of multiplexers in other dimensions^55–58^, our architecture can enable the corresponding chiral switching.

In summary, we have developed a topology-optimized EET architecture that emulates dynamic EP encirclement, utilizing an array of all-fiber devices to realize high-performance multidimensional asymmetric switching. This architecture replaces conventional couplers with multiplexers, effectively functioning as leaky devices while simultaneously reducing the 3-dB inherent loss and the number of active components (attenuators and amplifiers). Furthermore, we have implemented all-fiber multiplexers across multiple dimensions to accomplish high-performance asymmetric switching in polarization, mode, and phase. These all-fiber devices can be seamlessly integrated into optical communication systems. Notably, this work is the successful experimental demonstration of practical asymmetric OAM switching for the first time. Additionally, the proposed EET architecture demonstrates excellent compatibility with photonic integration platforms, significantly advancing the state-of-the-art in device miniaturization. These advancements establish a foundation for universal, flexible, and broadband multidimensional asymmetric switching, opening new opportunities in multidimensional non-Hermitian photonics exploration.

## Materials and Methods

### Fabrications

The all-fiber homemade (de)multiplexer is fabricated by using standard single-mode fiber and multi-mode fiber through a fused tapering technique. Simulation analysis is performed to determine the mode effective index at different taper ratios and to identify the phase matching points. The single-mode fiber undergoes pre-tapering to ensure that the effective index of its fundamental mode matches the target mode in the multi-mode fiber. Subsequently, both the pre-tapered single-mode fiber and multi-mode fiber are fusion tapered simultaneously using a hydrogen-oxygen flame, with real-time monitoring of the power at both output ports of the coupler to ensure maximum coupling efficiency for mode conversion. More details on the all-fiber device design and fabrication can be found in Supplementary Section S4.

### Measurements

For intensity profile measurement, the light emitted by the laser is passed through MUX, a non-Hermitian device and collimator, with the final output being received by the camera. PCs are added in front of and behind the MSC to control the polarization state and phase of the light. For interferogram measurement, the laser is divided into two homologous beams through an optical coupler, one of which passes through a MUX to generate a beam of corresponding mode, then undergoes asymmetric mode switching through a non-Hermitian device, and finally passes through a collimator. The other Gaussian beam passes through an optical attenuator and collimator, and the two beams interfere with each other through the beam splitter. The interferogram is received by the camera. The detailed measurement device diagram can be found in Supplementary Section S6.

## Supplementary information

Supplementary material

## Data Availability

All data are available in the main text or the supplementary materials.

## References

[1] El-Ganainy, R. et al. Non-Hermitian physics and PT symmetry. *Nat. Phys.* **14**, 11–19 (2018).

[2] Bender, C. M. & Boettcher, S. Real spectra in non-Hermitian Hamiltonians having *PT* symmetry. *Phys. Rev. Lett.* **80**, 5243–5246 (1998).

[3] Liu, T., Guo, C., Li, W. & Fan, S. Thermal photonics with broken symmetries. *eLight* **2**, 25 (2022).

[4] Ashida, Y., Gong, Z. P. & Ueda, M. Non-Hermitian physics. *Adv. Phys.* **69**, 249–435 (2020).

[5] Kawabata, K. et al. Symmetry and topology in non-Hermitian physics. *Phys. Rev. X* **9**, 041015 (2019).

[6] Kim, C. et al. Parity-time symmetry enabled ultra-efficient nonlinear optical signal processing. *eLight* **4**, 6 (2024). 38585278 10.1186/s43593-024-00062-w PMC10995095

[7] Zhong, Q. et al. Winding around non-Hermitian singularities. *Nat. Commun.* **9**, 4808 (2018). 30442951 10.1038/s41467-018-07105-0 PMC6237871

[8] Song, W. G. et al. Breakup and recovery of topological zero modes in finite non-Hermitian optical lattices. *Phys. Rev. Lett.* **123**, 165701 (2019). 31702358 10.1103/PhysRevLett.123.165701

[9] Zhou, H. Y. et al. Exceptional surfaces in *PT*-symmetric non-Hermitian photonic systems. *Optica* **6**, 190–193 (2019).

[10] Xu, Y. H. et al. Subwavelength control of light transport at the exceptional point by non-Hermitian metagratings. *Sci. Adv.* **9**, eadf3510 (2023). 37172089 10.1126/sciadv.adf3510 PMC10181182

[11] Choi, Y. et al. Observation of an anti-PT-symmetric exceptional point and energy-difference conserving dynamics in electrical circuit resonators. *Nat. Commun.* **9**, 2182 (2018). 29872042 10.1038/s41467-018-04690-y PMC5988699

[12] Lee, H. et al. Chiral exceptional point and coherent suppression of backscattering in silicon microring with low loss Mie scatterer. *eLight* **3**, 20 (2023).

[13] Miri, M. A. & Alù, A. Exceptional points in optics and photonics. *Science* **363**, eaar7709 (2019). 30606818 10.1126/science.aar7709

[14] Ding, K. et al. Emergence, coalescence, and topological properties of multiple exceptional points and their experimental realization. *Phys. Rev. X* **6**, 021007 (2016).

[15] Zhen, B. et al. Spawning rings of exceptional points out of Dirac cones. *Nature* **525**, 354–358 (2015). 26352476 10.1038/nature14889

[16] Yin, X. B. & Zhang, X. Unidirectional light propagation at exceptional points. *Nat. Mater.* **12**, 175–177 (2013). 23422707 10.1038/nmat3576

[17] Lin, Z. et al. Unidirectional Invisibility Induced by *PT*-symmetric periodic structures. *Phys. Rev. Lett.* **106**, 213901 (2011). 21699297 10.1103/PhysRevLett.106.213901

[18] Ramezani, H. et al. *PT*-symmetric talbot effects. *Phys. Rev. Lett.* **109**, 033902 (2012). 22861852 10.1103/PhysRevLett.109.033902

[19] Weimann, S. et al. Topologically protected bound states in photonic parity–time-symmetric crystals. *Nat. Mater.* **16**, 433–438 (2017). 27918567 10.1038/nmat4811

[20] Xu, H. et al. Topological energy transfer in an optomechanical system with exceptional points. *Nature* **537**, 80–83 (2016). 27454555 10.1038/nature18604

[21] Ergoktas, M. S. et al. Topological engineering of terahertz light using electrically tunable exceptional point singularities. *Science* **376**, 184–188 (2022). 35389774 10.1126/science.abn6528 PMC7612901

[22] Wiersig, J. Review of exceptional point-based sensors. *Photonics Res.* **8**, 1457–1467 (2020).

[23] Wiersig, J. Enhancing the sensitivity of frequency and energy splitting detection by using exceptional points: application to microcavity sensors for single-particle detection. *Phys. Rev. Lett.* **112**, 203901 (2014).

[24] Hodaei, H. et al. Enhanced sensitivity at higher-order exceptional points. *Nature* **548**, 187–191 (2017). 28796201 10.1038/nature23280

[25] Hokmabadi, M. P. et al. Non-Hermitian ring laser gyroscopes with enhanced Sagnac sensitivity. *Nature* **576**, 70–74 (2019). 31802015 10.1038/s41586-019-1780-4

[26] Mao, W. B. et al. Exceptional–point–enhanced phase sensing. *Sci. Adv.* **10**, eadl5037 (2024). 38579005 10.1126/sciadv.adl5037 PMC10997194

[27] Xiao, Z. C. et al. Enhanced sensing and nondegraded thermal noise performance based on *PT*-symmetric electronic circuits with a sixth-order exceptional point. *Phys. Rev. Lett.* **123**, 213901 (2019). 31809159 10.1103/PhysRevLett.123.213901

[28] Chen, W. J. et al. Exceptional points enhance sensing in an optical microcavity. *Nature* **548**, 192–196 (2017). 28796206 10.1038/nature23281

[29] Zhao, W. Z. et al. Exceptional points induced by unidirectional coupling in electronic circuits. *Nat. Commun.* **15**, 9907 (2024). 39548062 10.1038/s41467-024-53929-4 PMC11568309

[30] Kononchuk, R. et al. Exceptional-point-based accelerometers with enhanced signal-to-noise ratio. *Nature* **607**, 697–702 (2022). 35896648 10.1038/s41586-022-04904-w

[31] Doppler, J. et al. Dynamically encircling an exceptional point for asymmetric mode switching. *Nature* **537**, 76–79 (2016). 27454554 10.1038/nature18605

[32] Hassan, A. U. et al. Dynamically encircling exceptional points: exact evolution and polarization state conversion. *Phys. Rev. Lett.* **118**, 093002 (2017). 28306295 10.1103/PhysRevLett.118.093002

[33] Li, A. D. et al. Hamiltonian hopping for efficient chiral mode switching in encircling exceptional points. *Phys. Rev. Lett.* **125**, 187403 (2020). 33196255 10.1103/PhysRevLett.125.187403

[34] Li, A. D. et al. Riemann-encircling exceptional points for efficient asymmetric polarization-locked devices. *Phys. Rev. Lett.* **129**, 127401 (2022). 36179197 10.1103/PhysRevLett.129.127401

[35] Liu, Q. J. et al. Efficient mode transfer on a compact silicon chip by encircling moving exceptional points. *Phys. Rev. Lett.* **124**, 153903 (2020). 32357032 10.1103/PhysRevLett.124.153903

[36] Wei, Y. X. et al. Anti-parity-time symmetry enabled on-chip chiral polarizer. *Photonics Res.* **10**, 76 (2022).

[37] Nasari, H. et al. Observation of chiral state transfer without encircling an exceptional point. *Nature* **605**, 256–261 (2022). 35546193 10.1038/s41586-022-04542-2

[38] Qi, H. X. et al. Dynamically encircling exceptional points in different Riemann sheets for orbital angular momentum topological charge conversion. *Phys. Rev. Lett.* **132**, 243802 (2024). 38949371 10.1103/PhysRevLett.132.243802

[39] Wang, S. W. et al. An ultralow crosstalk and broadband subwavelength grating-assisted chiral mode converter by encircling exceptional points. *Appl. Phys. Lett.* **123**, 241103 (2023).

[40] Yu, F. et al. General rules governing the dynamical encircling of an arbitrary number of exceptional points. *Phys. Rev. Lett.* **127**, 253901 (2021). 35029432 10.1103/PhysRevLett.127.253901

[41] Zhang, X. L. & Chan, C. T. Dynamically encircling exceptional points in a three-mode waveguide system. *Commun. Phys.* **2**, 63 (2019).

[42] Feng, Z. Y. & Sun, X. K. Harnessing dynamical encircling of an exceptional point in anti-PT-symmetric integrated photonic systems. *Phys. Rev. Lett.* **129**, 273601 (2022). 36638290 10.1103/PhysRevLett.129.273601

[43] Li, A. D. et al. Exceptional points and non-Hermitian photonics at the nanoscale. *Nat. Nanotechnol.* **18**, 706–720 (2023). 37386141 10.1038/s41565-023-01408-0

[44] Shu, X. Q. et al. Fast encirclement of an exceptional point for highly efficient and compact chiral mode converters. *Nat. Commun.* **13**, 2123 (2022). 35440654 10.1038/s41467-022-29777-5 PMC9018827

[45] Khurgin, J. B. et al. Emulating exceptional-point encirclements using imperfect (leaky) photonic components: asymmetric mode-switching and omni-polarizer action. *Optica* **8**, 563–569 (2021).

[46] Choi, Y. S. et al. Broadband optical nonreciprocity by emulation of nonlinear non-Hermitian time-asymmetric loop. *Commun. Phys.* **7**, 263 (2024).

[47] Kim, S. & Bahl, G. Exceptional behaviour without exceptional effort. *Nat. Photonics* **15**, 556–557 (2021).

[48] Winzer, P. J. Making spatial multiplexing a reality. *Nat. Photonics* **8**, 345–348 (2014).

[49] Li, K. et al. Fiber–chip-fiber mode/polarization/wavelength transmission and processing with few-mode fiber, (de)Multiplexing SiO_2_ chip and ROADM Si chip. *Laser Photonics Rev.* **18**, 2300489 (2024).

[50] Zhou, W. et al. All-fiber function devices for twisted lights. *Opt. Express* **31**, 43438–43448 (2023). 38178437 10.1364/OE.504437

[51] Chuang, S. L. A coupled mode formulation by reciprocity and a variational principle. *J. Lightwave Technol.* **5**, 5–15 (1987).

[52] Richardson, D. J., Fini, J. M. & Nelson, L. E. Space-division multiplexing in optical fibres. *Nat. Photonics* **7**, 354–362 (2013).

[53] Chang, W. J. et al. Ultra-compact mode (de) multiplexer based on subwavelength asymmetric Y-junction. *Opt. Express* **26**, 8162–8170 (2018). 29715785 10.1364/OE.26.008162

[54] He, Y. et al. Ultra-compact and broadband silicon polarizer employing a nanohole array structure. *Opt. Lett.* **46**, 194–197 (2021). 33448986 10.1364/OL.403819

[55] He, C., Shen, Y. & Forbes, A. Towards higher-dimensional structured light. *Light Sci. Appl.* **11**, 205 (2022). 35790711 10.1038/s41377-022-00897-3 PMC9256673

[56] Shen, Y. et al. Optical skyrmions and other topological quasiparticles of light. *Nat. Photonics* **18**, 15–25 (2024).

[57] Shen, Y., Hou, Y., Papasimakis, N. & Zheludev, N. I. Supertoroidal light pulses as electromagnetic skyrmions propagating in free space. *Nat. Commun.* **12**, 5891 (2021). 34625539 10.1038/s41467-021-26037-w PMC8501108

[58] Shen, Y. et al. Optical vortices 30 years on: OAM manipulation from topological charge to multiple singularities. *Light Sci. Appl.* **8**, 90 (2019). 31645934 10.1038/s41377-019-0194-2 PMC6804826

