# Supplementary material for: Exceptional-point-encirclement emulation tailoring: multidimensional asymmetric switching of all-fiber devices
Source: Light Sci Appl. 2026 Jan 1;15:8. doi: 10.1038/s41377-025-02144-x (PMC12756234; doi:10.1038/s41377-025-02144-x)
Supplement: Supplementary file 1 — Supplementary material [file 41377_2025_2144_MOESM1_ESM.docx]

Supplementary material for “Exceptional-point-encirclement emulation tailoring: multidimensional asymmetric switching of all-fiber devices”

**Kang Li ^1, 2, 3, #^, Yuchen Zhang ^1, 2, 3, #^, Siwei Wang ^1, 2, 3^, and Jian Wang ^1, 2, 3^,***

^1^ Wuhan National Laboratory for Optoelectronics and School of Optical and Electronic Information, Huazhong University of Science and Technology, Wuhan 430074, Hubei, China.

^2^ Hubei Optical Fundamental Research Center, Wuhan 430074, China

^3^ Optics Valley Laboratory, Wuhan 430074, Hubei, China

^#^ These authors contributed equally.

* Corresponding author: jwang@hust.edu.cn.

Contents

[S1: Asymmetric switching through exception point encirclement 1](#_Toc200801283)

[S2: Crosstalk and minimum transformation loss with different coupling efficiency 4](#_Toc200801284)

[S3: Equation derivation for the reverse transmission matrix 6](#_Toc200801285)

[S4: All-fiber device design and fabrication 7](#_Toc200801286)

[S5: Measurement setup of asymmetric switching for various spatial/phase lights 11](#_Toc200801287)

[S6: Measurement setup for the intensity profiles and interferograms 13](#_Toc200801288)

[S7: Performance analysis of polarization (de)multiplexer 14](#_Toc200801289)

[S8: Optimized topological architecture in compact photonic integrated circuit 16](#_Toc200801290)

[Reference 19](#_Toc200801291)

# S1: Asymmetric switching through exception point encirclement

The general form of the non-Hermitian system matrix is given:

$$H=\left(\begin{matrix} \beta_{1}-i\gamma_{1} & \kappa\\ \kappa& \beta_{2}-i\gamma_{2} \end{matrix}\right)$$

To simplify the Hamiltonian matrix, we set *β*_1_ = -*β*_2_ ≡ *β*_0_ and *γ*_2_ = 0, and divide the entire matrix by *κ*, resulting in a reduced non-Hermitian system matrix:

$$H_{1}=\left(\begin{matrix} \beta_{0}/\kappa-i\gamma_{1}/\kappa& 1 \\ 1 & -\beta_{0}/\kappa\end{matrix}\right)$$

Figs. S1(a, c) and S1(b, d) display the real and imaginary parts of the eigenvalues of *H*_1_, respectively. Based on these two Riemann surfaces, we illustrate the mechanism of asymmetric switching induced by exceptional point (EP) encirclement. Solid (dashed) trajectories correspond to the input state being the even (odd) mode, represented by the eigenstates [1,1] and [1,-1], respectively.

Figs. S1(a, b) illustrate the case of clockwise encirclement. The two different eigenstates originate from the starting point and evolve along the blue and red Riemann surfaces. From the imaginary-part Riemann surface, it can be seen that the red sheet corresponds to high loss, while the blue sheet indicates low loss. As a result, the odd mode evolves along the low-loss blue Riemann surface, undergoes EP encirclement, and transforms into the even mode upon crossing the branch cut on the real-part surface. In contrast, the even mode, which traverses the high-loss red Riemann sheet, experiences strong attenuation and undergoes nonadiabatic transitions (NATs), switching its trajectory to the blue Riemann sheet. It then continues along this sheet and eventually maintains its original even-mode identity after crossing the real-part branch cut. Therefore, regardless of the initial state, the output mode after clockwise encirclement is always the even mode.


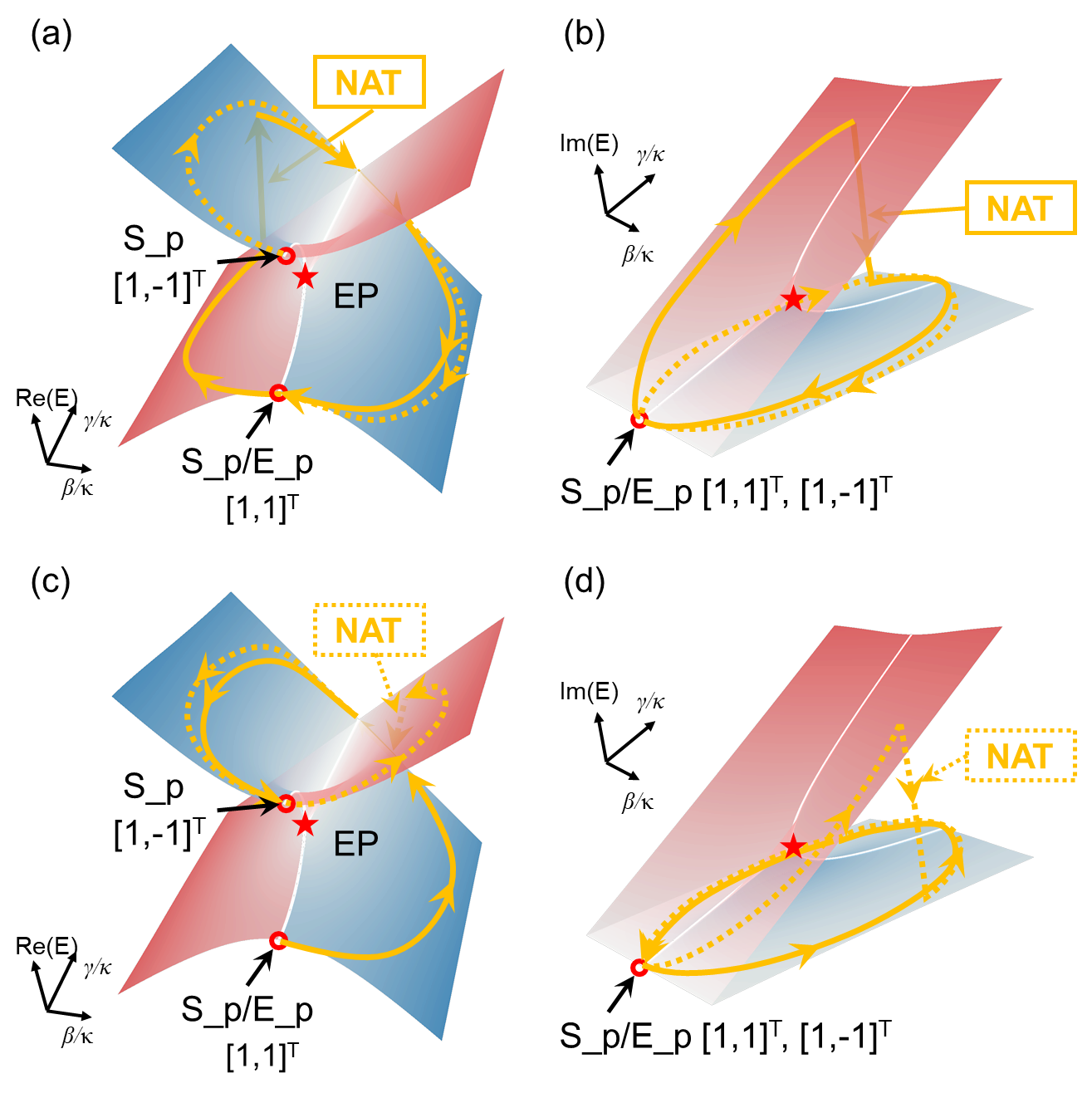


**Fig. S1** Asymmetric switching based on EP encirclement. (a), (b) correspond to clockwise encirclement, while (c), (d) correspond to counterclockwise encirclement. (a), (c) show the real parts of the eigenvalues, and (b), (d) show the imaginary parts. The encirclement begins at the starting point S_p and ends at the endpoint E_p, where the two eigenstates [1,1] and [1, -1] represent the even and odd modes of the system, respectively. S_p: starting point; E_p: ending point.

Figs. S1(c, d) present the counterclockwise encirclement case. Similarly, the two distinct eigenstates evolve along the red and blue Riemann surfaces. The even mode follows the blue, low-loss sheet, completes the EP encirclement, and transforms into the odd mode upon crossing the real-part branch cut. Meanwhile, the odd mode suffers significant attenuation on the red, high-loss sheet and undergoes NATs, switching to the blue Riemann surface. It then maintains its original identity as an odd mode after crossing the branch cut. Consequently, for counterclockwise encirclement, the output mode is consistently the odd mode, regardless of the input state.

# S2: Crosstalk and minimum transformation loss with different coupling efficiency

Fig. S2 shows the crosstalk and the minimum transformation loss with different coupling efficiency *κ*^2^ of the coupler in the conventional non-adiabatic evolution architecture. It can be observed that when *κ*^2^ is 0.5, the crosstalk values of the output modes for inputs from both the left and right sides are similar, indicating the realization of asymmetric switching. However, this conventional architecture suffers from a non-negligible 3 dB loss.


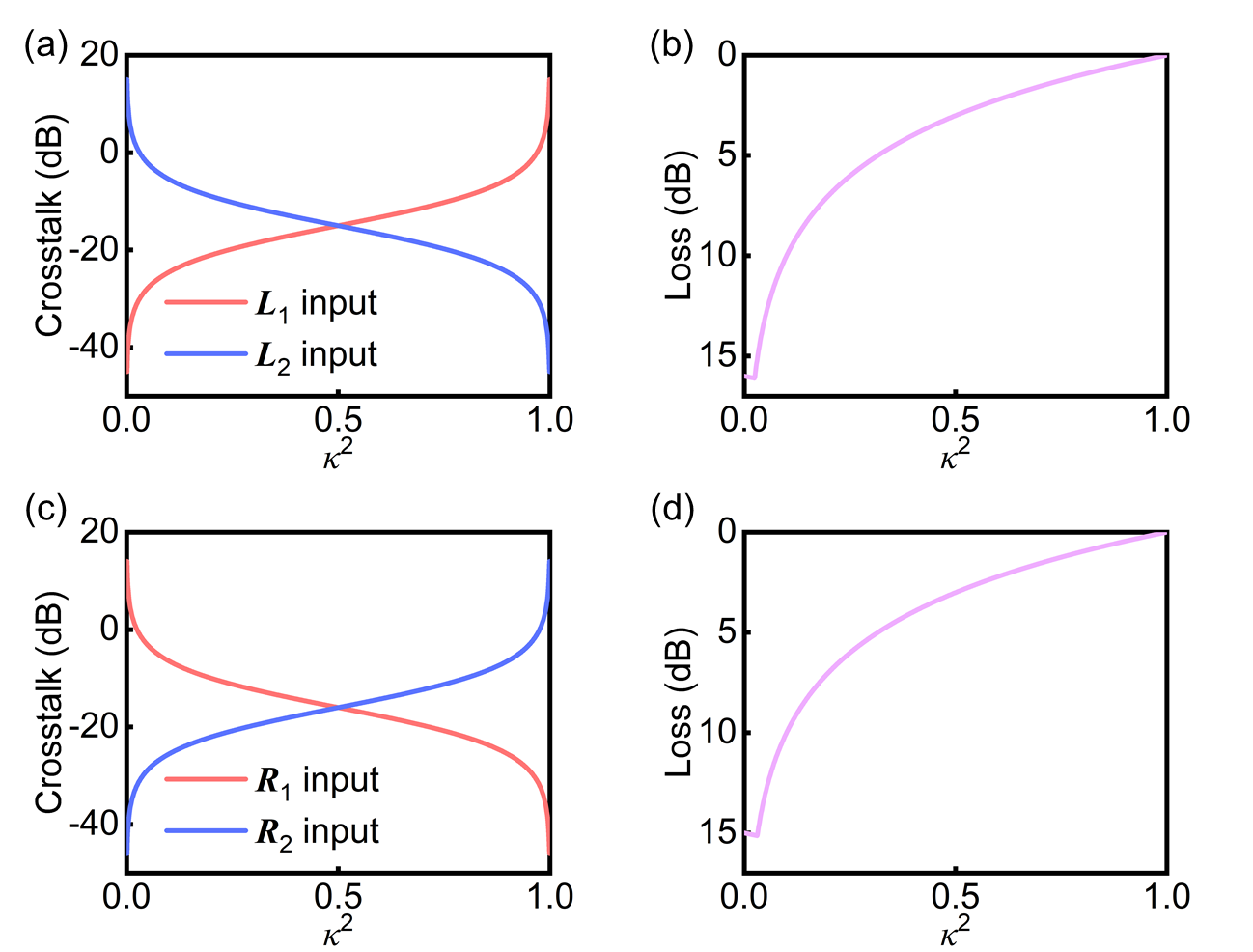


**Fig. S2** Transmission performance of conventional non-adiabatic evolution architecture. (a), (c) Crosstalk and (b), (d) minimum transformation loss with different coupling efficiency *κ*^2^, when light is injected in different ports at (a), (b) left side and (c), (d) right side.

Fig. S3 shows the crosstalk and the minimum transformation loss with different coupling efficiency *κ*^2^ of the (de)multiplexer in the topology-optimized architecture. The coupling efficiencies of the two multiplexers are set to *κ*^2^ and *κ*^2^ + 0.01, respectively. It can be observed that, regardless of whether the input is from the left or the right, the minimum transformation loss decreases as the coupling efficiency of the multiplexer increases. Therefore, as the coupling efficiency approaches one, this architecture can achieve lossless transformation. Moreover, since the output crosstalk of the device gradually decreases with increasing *κ*^2^, the optimized architecture can maintain low modal crosstalk while realizing lossless transmission.


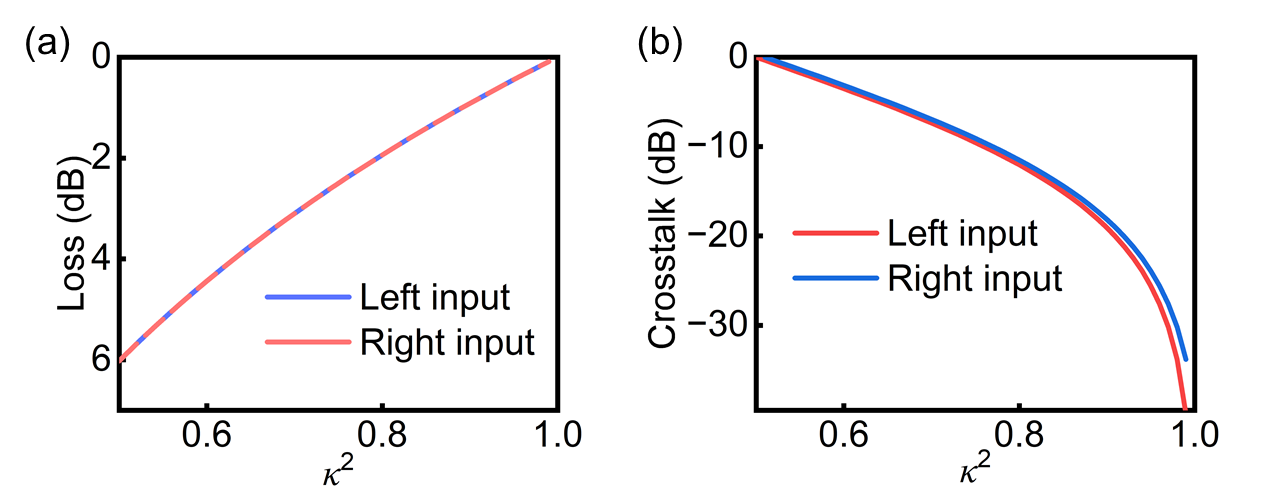


**Fig. S3** Transmission performance of topology optimization architecture. (a) Minimum transformation loss and (b) crosstalk with different coupling efficiency *κ*^2^ in (de)multiplexer.

# S3: Equation derivation for the reverse transmission matrix

We evaluate the performance of this architecture through its reverse transmission matrix, as shown in Fig. S4. The forward transmission matrix is as follows:

$$\overleftarrow{T_{Top}}=T_{\#2\to\#3\to\#1}=\left[\begin{matrix} T_{\overleftarrow{L_{1}R_{1}}} & T_{\overleftarrow{L_{2}R_{1}}} \\ T_{\overleftarrow{L_{1}R_{2}}} & T_{\overleftarrow{L_{2}R_{2}}} \end{matrix}\right]=\left[\begin{matrix} T_{\overleftarrow{L_{1}M_{1}R_{1}}}+T_{\overleftarrow{L_{1}M_{2}R_{1}}} & T_{\overleftarrow{L_{2}M_{1}R_{1}}}+T_{\overleftarrow{L_{2}M_{2}R_{1}}} \\ T_{\overleftarrow{L_{1}M_{1}R_{2}}}+T_{\overleftarrow{L_{1}M_{2}R_{2}}} & T_{\overrightarrow{L_{2}M_{1}R}_{2}}+T_{\overrightarrow{L_{2}M_{2}R}_{2}} \end{matrix}\right]$$

Due to the huge loss of optical attenuator (*γ*_6_ > 60 dB), the transmission of experiencing optical attenuator can be ignored to further simplify the transmission matrix $\overleftarrow{T_{Top}}$.

$$\begin{matrix} \overleftarrow{T_{Top}}=T_{\#2\to\#3\to\#1}=\left[\begin{matrix} T_{\overleftarrow{L_{1}R_{1}}} & T_{\overleftarrow{L_{2}R_{1}}} \\ T_{\overleftarrow{L_{1}R_{2}}} & T_{\overleftarrow{L_{2}R_{2}}} \end{matrix}\right]\approx\left[\begin{matrix} T_{\overleftarrow{L_{1}M_{2}R_{1}}} & T_{\overleftarrow{L_{2}M_{2}R_{1}}} \\ T_{\overleftarrow{L_{1}M_{2}R_{2}}} & T_{\overrightarrow{L_{2}M_{2}R}_{2}} \end{matrix}\right] \\ =\left[\begin{matrix} 10*\lg(\kappa_{1}^{2})+10*\lg(1-\kappa_{2}^{2})+\gamma_{5} & 10*\lg(1-\kappa_{1}^{2})+10*\lg(1-\kappa_{2}^{2})+\gamma_{5} \\ 20*\lg(\kappa_{1}^{2})+\gamma_{5} & 10*\lg(\kappa_{1}^{2})+10*\lg(1-\kappa_{1}^{2})+\gamma_{5} \end{matrix}\right] \end{matrix}$$

Similarly, the amplifier is not used, and we select commonly used MSC performance values, specifically: *γ*_5_ = 0 dB, *κ*_1_^2^ = 0.97, *κ*_2_^2^ = 0.98. The matrix can be further expressed as:

$$\overleftarrow{T_{Top}}=\left[\begin{matrix} -17.12 & -32.22 \\ -0.22 & -15.36 \end{matrix}\right]$$

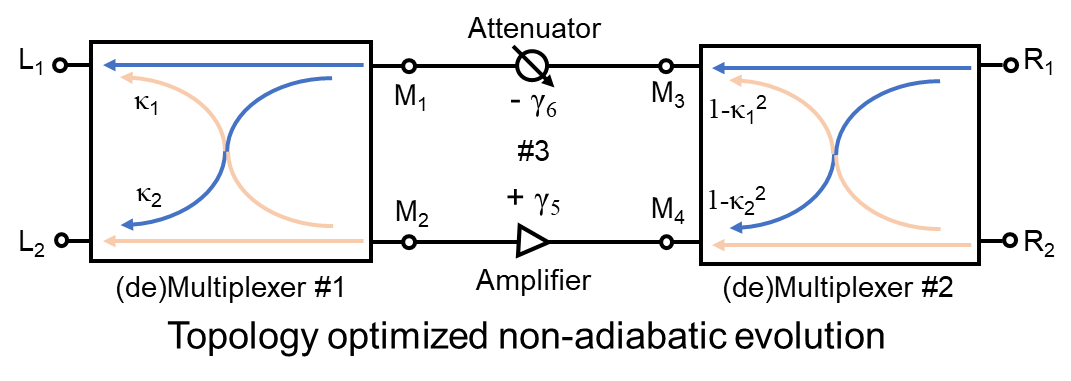


**Fig. S4** Topology-optimized non-adiabatic evolution when light is injected in right ports.

# S4: All-fiber device design and fabrication

We use single-mode fiber (SMF) and multi-mode fiber (MMF) to fabricate all-fiber mode-selective coupler (MSC) employing fiber fused tapering method. The theoretical refractive index profile of the SMF and the measured refractive index profile of the MMF are shown in Fig. S5.


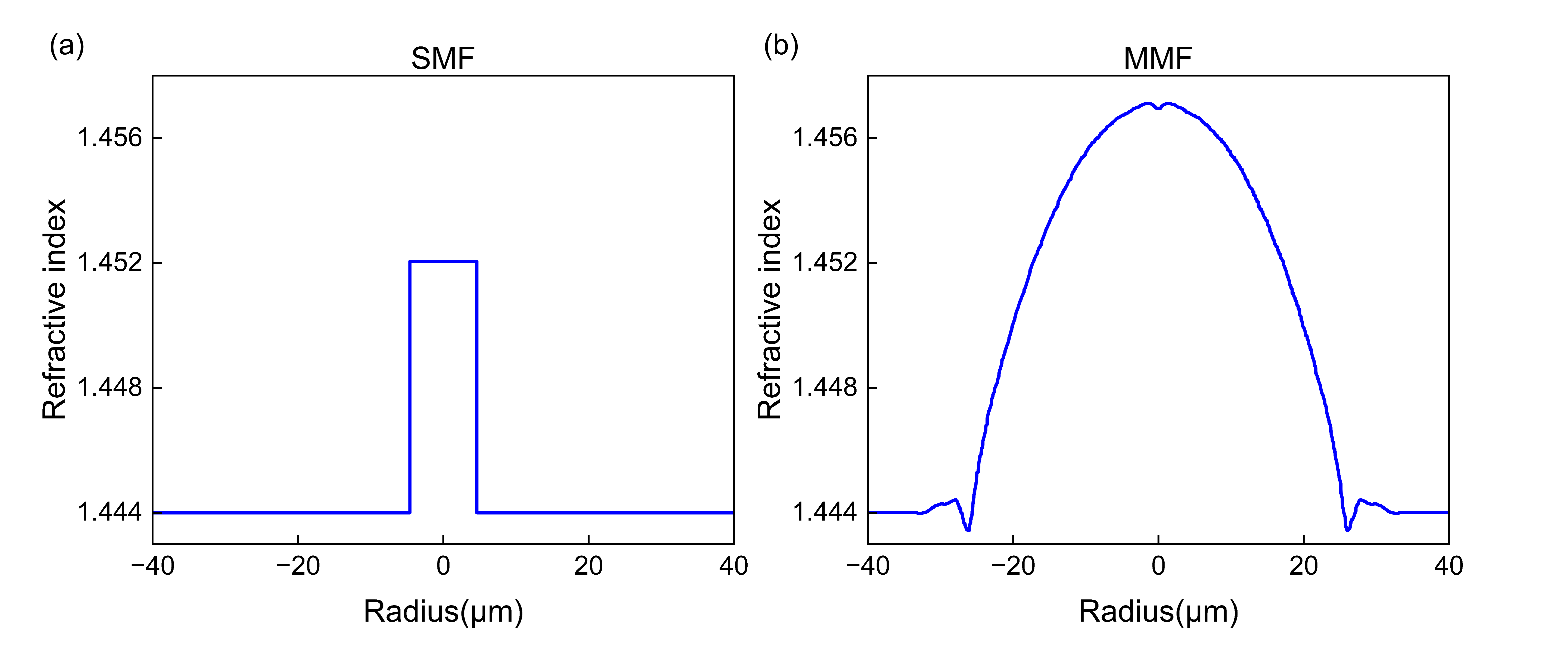


**Fig. S5** Refractive index profile of fibers. (a) The theoretical refractive index profile of the SMF and (b) the measured refractive index profile of the MMF.

The SMF support $HE_{11}^{e\text{∕}o}$ mode, whereas the MMF we use can support more than 100 modes, with the first-order mode group encompassing $HE_{21}^{e\text{∕}o}$, $TM_{01}$ and $TE_{01}$, and the second-order mode group encompassing $EH_{11}^{e\text{∕}o}$, $HE_{12}^{e\text{∕}o}$ and $HE_{31}^{e\text{∕}o}$. Given that higher-order radial modes are not within the scope of our requirements, $HE_{12}^{e\text{∕}o}$ mode is not considered in this paper.

Fig. S6 illustrates the simulated variation of the effective index (n_eff_) of HE_11_ mode in SMF and the TM_01_, TE_01_, HE_21_, EH_11_ and HE_31_ modes in MMF as a function of the taper ratio. Drawing from coupled-mode theory, coupling occurs exclusively when the effective refractive indices of distinct modes in two fibers are similar. We can discern that reveals that the n_eff_ of SMF does not align with that of the high-order modes within MMF, necessitating a pre-tapering process of the SMF to fulfill the phase-matching condition. The thick red (SMF_1_) and gray (SMF_2_) solid lines represent the SMF after pre-tapering, which are necessary for the fabrication of first-order and second-order mode couplers, respectively. The red dots indicate the phase-matching points for different modes. Following pre-treatment, we proceed with the fusion tapering of SMF and MMF to attain efficient mode coupling. The inset delineates the coupling of the LP_01_ mode from SMF_1_ into LP_11_ and OAM_11_ modes in MMF, and the LP_01_ mode from SMF_2_ into LP_21_ and OAM_21_ modes in MMF. It is worth noting that LP and OAM modes are capable of undergoing mode converting in MMF. Mode conversion can be achieved by using polarization controllers (PCs) in SMF and MMF to adjust the phase difference and proportion of different vector modes within the mode groups. The synthesis formulas for LP and OAM modes in our couplers are as follows:

$$LP_{11}^{a\text{∕}b}=TE_{01}\pm HE_{21}$$

$$LP_{21}^{a\text{∕}b}=EH_{11}\pm HE_{31}$$

$$OAM_{\pm11}=HE_{21}^{e}\pm iHE_{21}^{o}$$

$$OAM_{\pm21}=HE_{31}^{e}\pm iHE_{31}^{o}$$

The superscripts 'a' and 'b' for LP modes represent two orthogonal spatial states, while the superscripts 'e' and 'o' for HE modes represent the degenerate odd and even modes, respectively. Regarding the determination of whether the fabricated coupler is for the LP or OAM mode, it is crucial to observe the light output from MMF during the fabrication process, and conduct repeated experiments to determine the parameters.

Furthermore, this method theoretically supports the generation of 3-4 order modes. For simplicity, LP_31_ and LP_41_ are used here to represent the 3rd and 4th order modes. Fig. S7 shows simulated effective index of mode in SMF (LP_01_) and higher order modes in MMF (LP_31_ and LP_41_) as functions of fiber taper ratio. However, in the actual experimental process, we found that when the order reaches 3, the loss of the coupler will be so large that it cannot be ignored, exceeding 5dB. Therefore, higher order mode switching is not shown in the manuscript. In the future, process parameters can be optimized or specially designed MMF can be used to achieve chiral switching of higher order modes.


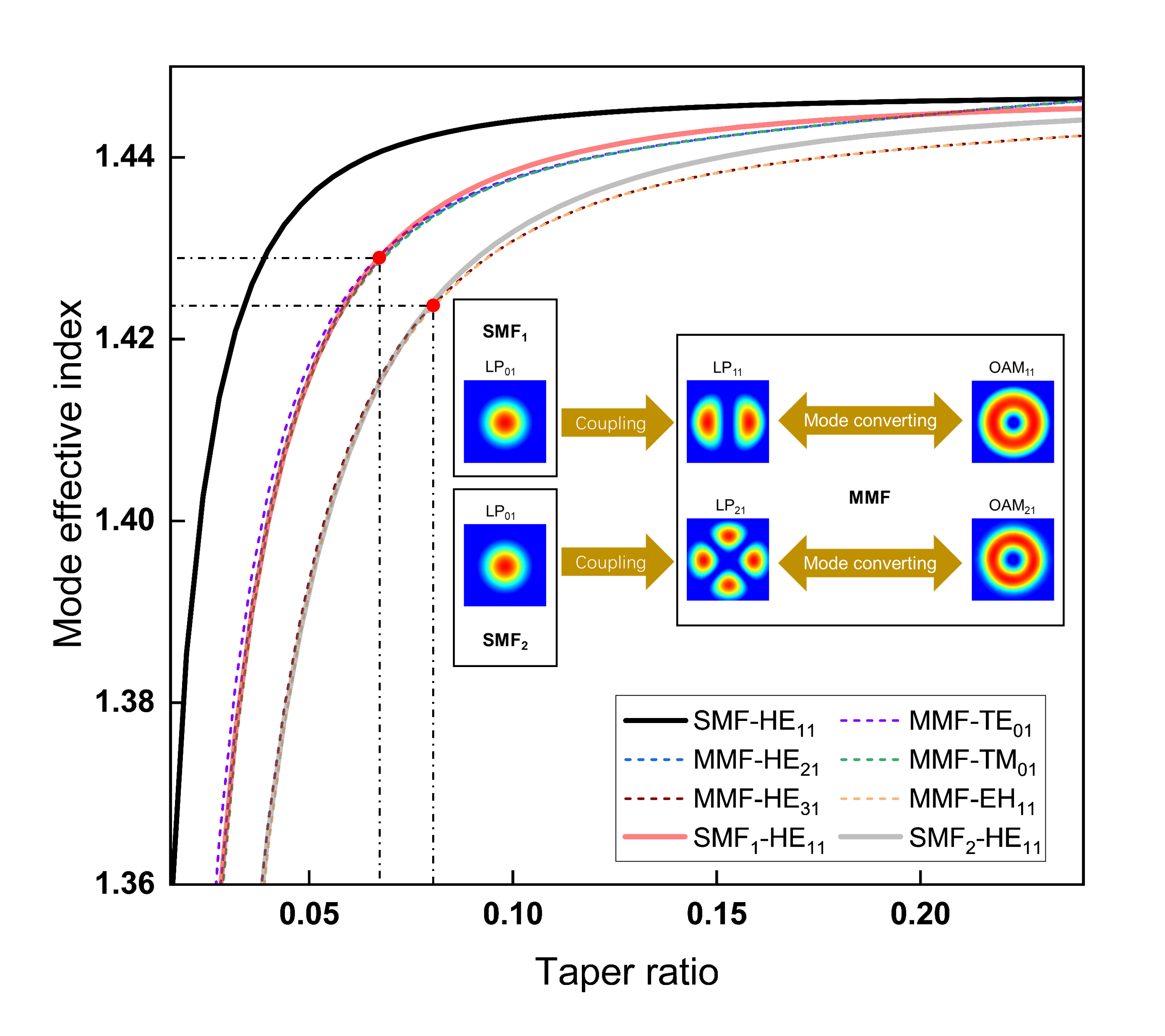


**Fig. S6** Simulated effective index of different modes in SMF and MMF as functions of fiber taper ratio and phase-matching point of the mode-selective couplers.


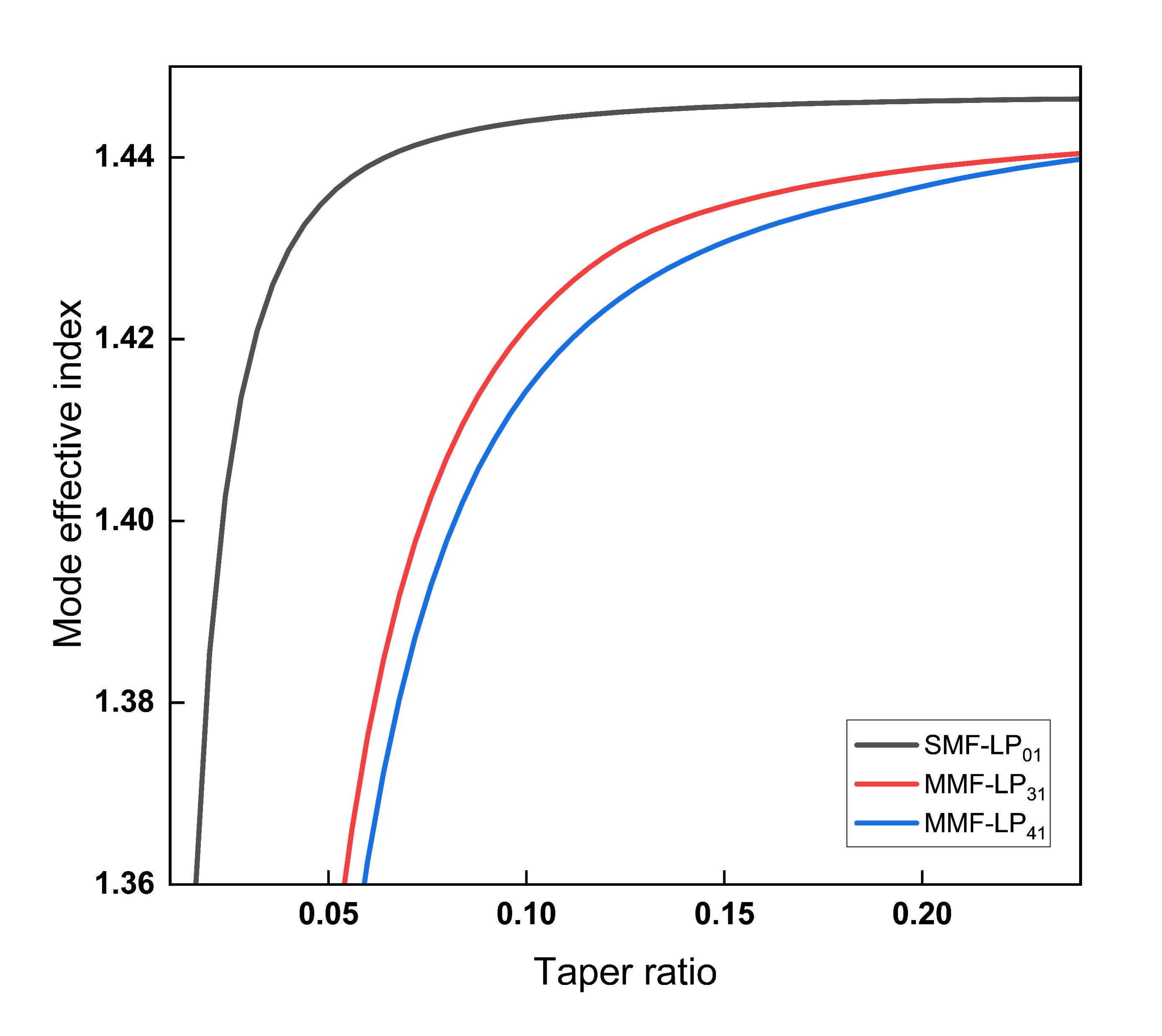


**Fig. S7** Simulated effective index of LP_01_ in SMF and higher order modes (LP_31_, LP_41_) in MMF as functions of fiber taper ratio.

# S5: Measurement setup of asymmetric switching for various spatial/phase lights

Figs. S8(a) and S9(a) depict the measurement setup of asymmetric switching for various spatial and phase lights, respectively. Due to the limited visibility of the fibers, Figs. S8(b) and S9(b) provide corresponding schematic architectures to clarify the experimental setup.


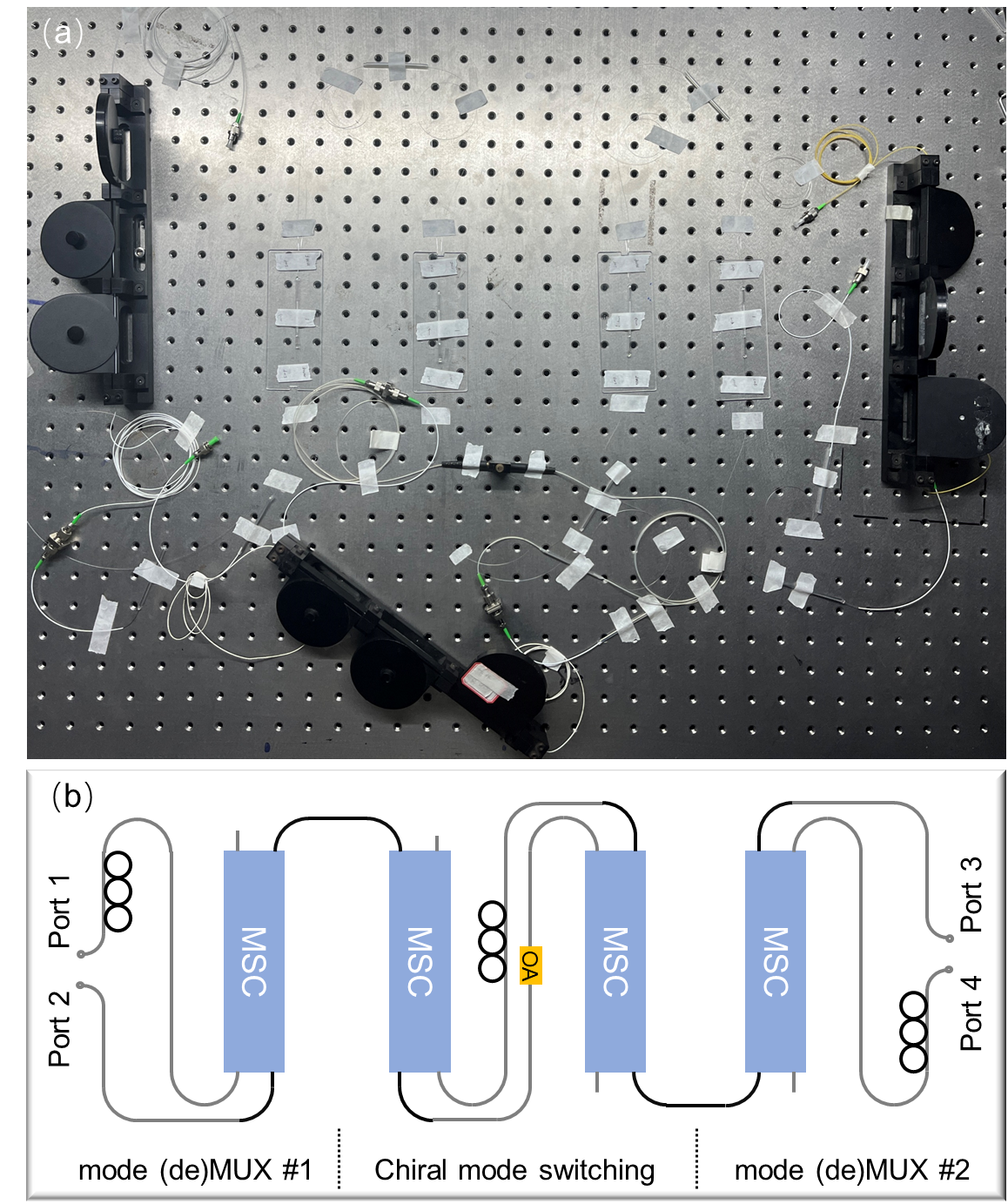


**Fig. S8** Measurement setup of asymmetric switching for various spatial lights. **(a)** Photos. **(b)** Architecture.


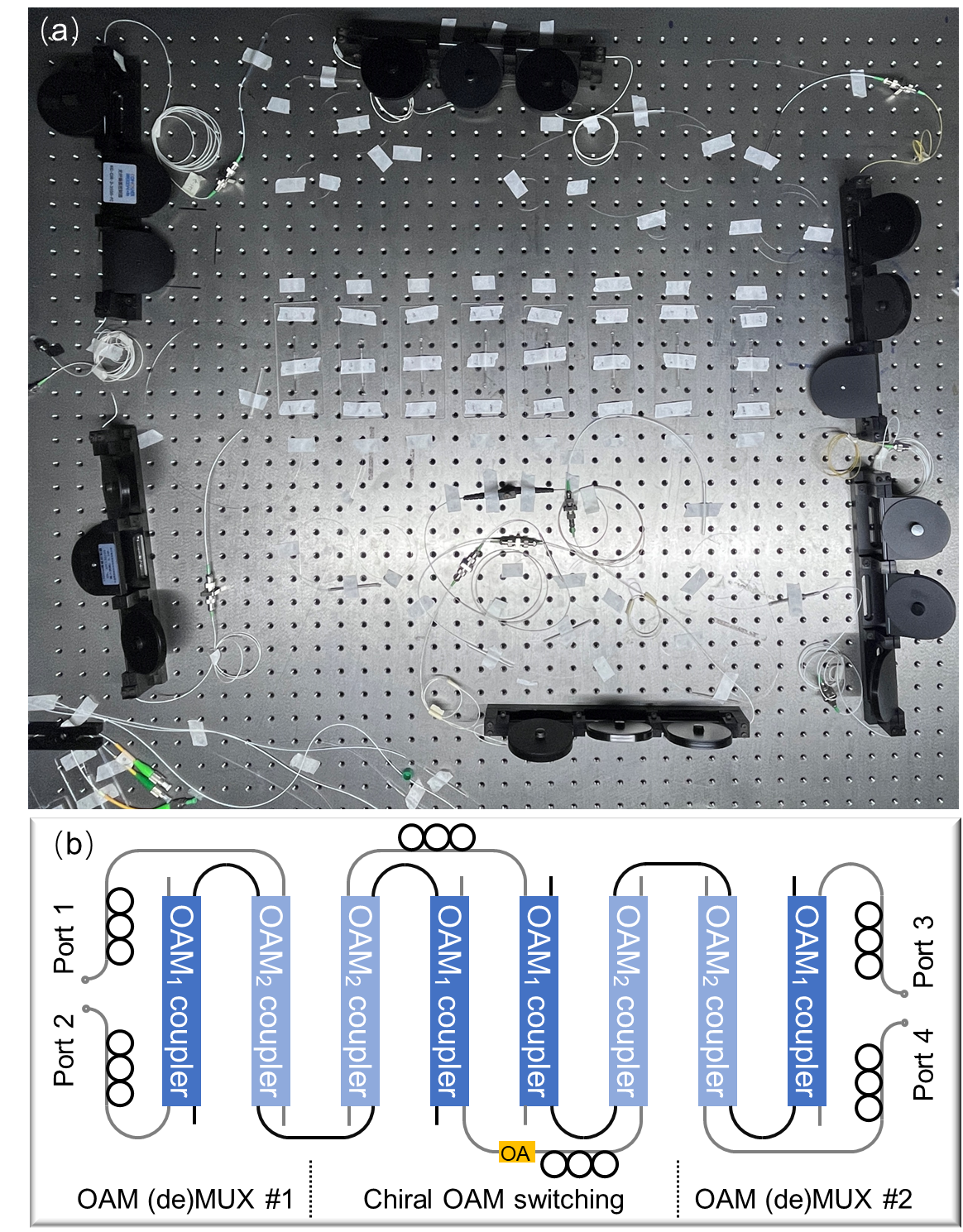


**Fig. S9** Measurement setup of asymmetric switching for various phase lights. **(a)** Photos. **(b)** Architecture.

# S6: Measurement setup for the intensity profiles and interferograms

Fig. S10 shows the measurement setup for the (a) intensity profiles and (b) interferograms. For intensity profiles, the light emitted by the laser is passed through MSC, non-Hermitian device (nHD) and collimator, with the final output being received by the camera. PCs are added in front of and behind the MSC to control the polarization state and phase of the light. For interferograms, the laser is divided into two homologous beams through an optical coupler (OC), one of which passes through an MSC to generate a beam of corresponding mode, then undergoes asymmetric mode conversion through non-Hermitian device, and finally passes through a collimator. The other Gaussian beam passes through an optical attenuator and collimator, and the two beams interfere with each other through a beam splitter (BS). The interferogram is received by the camera.


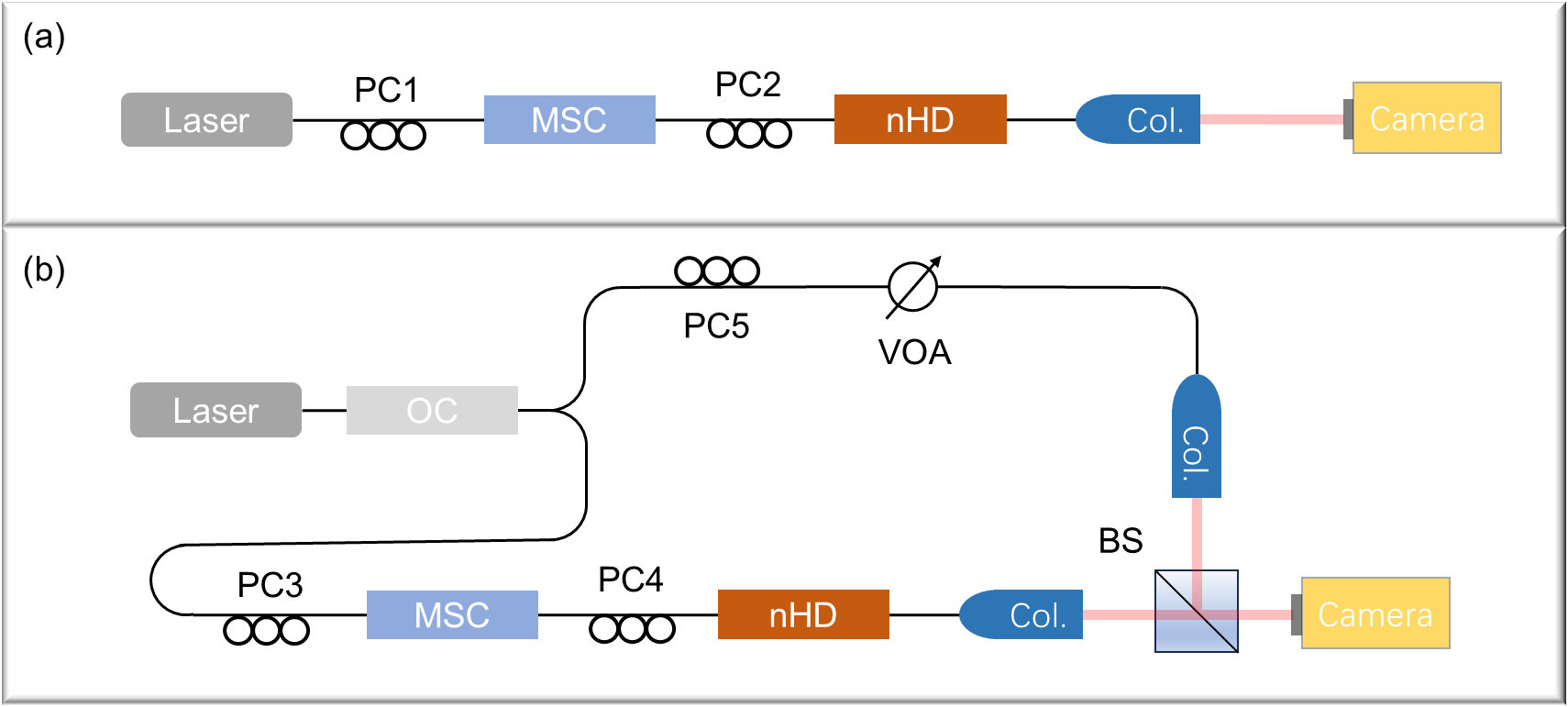


**Fig. S10** Measurement setups for mode field characterization in MMF. (a) Intensity profiles and (b) interferograms. OC: optical coupler; PC: polarization controller; MSC: mode-selective coupler; nHD: non-Hermitian device; Col.: collimator; VOA: variable optical attenuator; BS: beam splitter.

# S7: Performance analysis of polarization (de)multiplexer


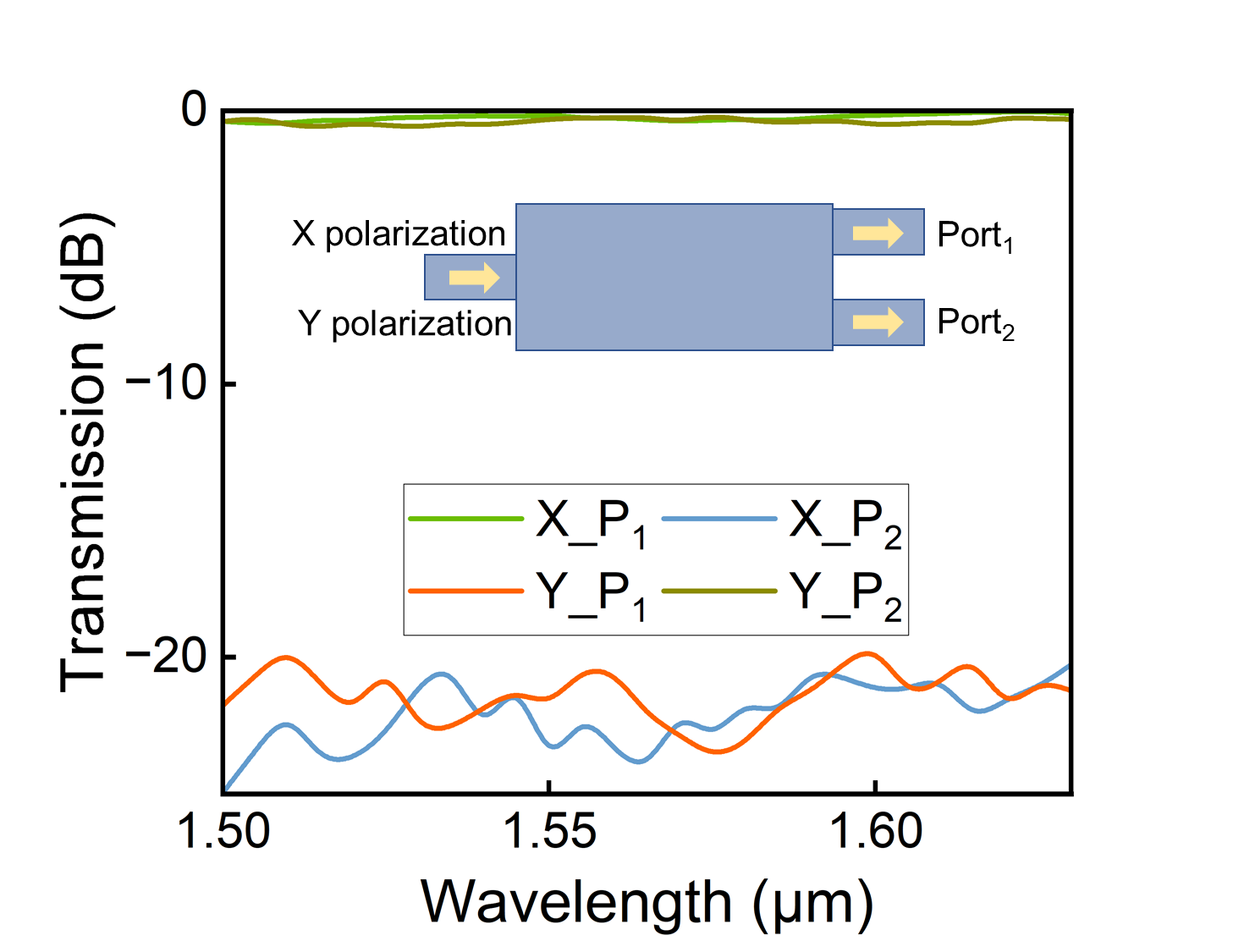


**Fig. S11** Measured transmission spectra of the loss and crosstalk of a polarization (de) multiplexer. The inset is a schematic diagram of the tested device.


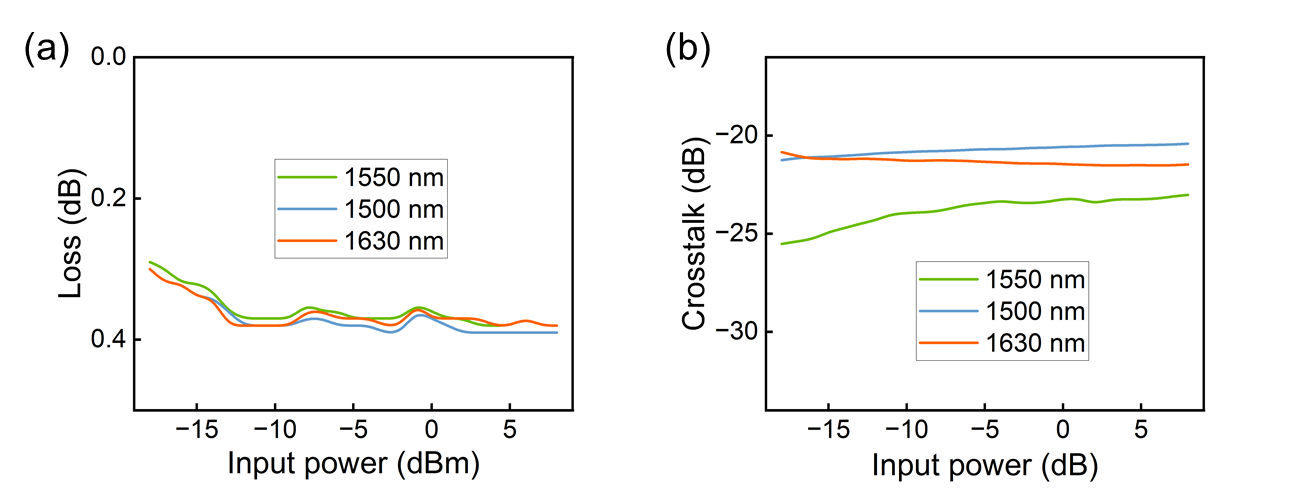


**Fig. S12** Measured performances of polarization (de)multiplexer at different input powers. (a) Loss and (b) crosstalk.

Fig. S11 shows the transmission spectra at the two demultiplexing ports when two polarizations are input into the multiplexing port of the polarization (de)multiplexer. The measured results demonstrate that the polarization (de)multiplexer exhibits excellent performance, with insertion loss below 0.4 dB and crosstalk below -20 dB across the 1.50-1.63 μm wavelength range. Additionally, Fig. S12 presents the variations in insertion loss and polarization crosstalk at wavelengths of 1500 nm, 1550 nm, and 1630 nm, as the input power changes from -18 dBm to 8 dBm. It can be observed that the fluctuation in insertion loss remains within 0.1 dB, while the variation in polarization crosstalk stays within 2.5 dB. These results indicate that the performance of the polarization multiplexer is largely unaffected by changes in input power.

# S8: Optimized topological architecture in compact photonic integrated circuit


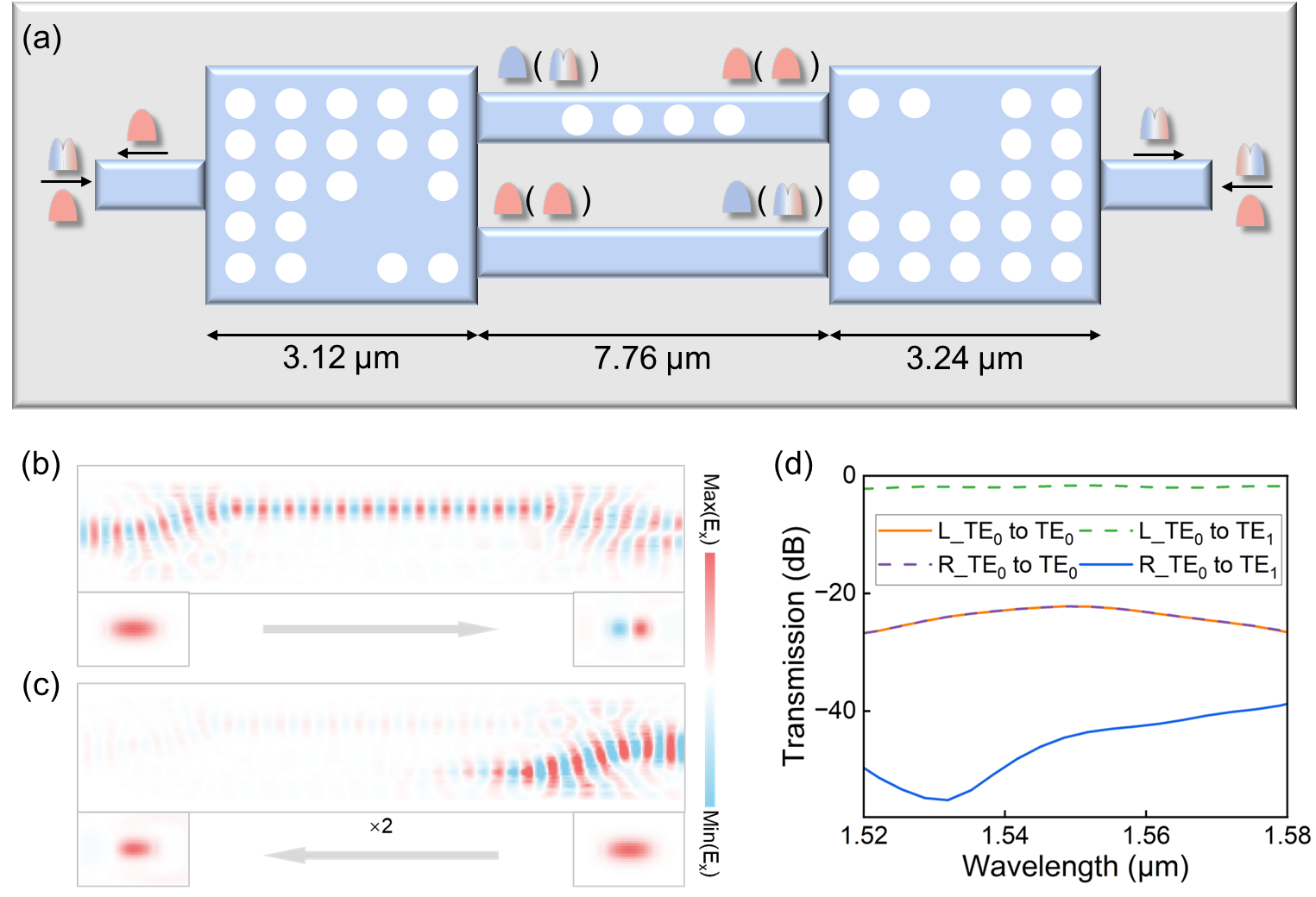


**Fig. S13** Compact silicon device based on the optimized topological architecture. (a) Structure diagram. (b), (c) Simulated field distributions at 1.55 µm, when the incident mode is injected from (b) the left side and (c) the right side. (d) Simulated transmission spectra of TE_0_/TE_1_ modes at output ports.

Figs. S13(a) depicts the compact photonic integrated circuit (PIC) based on the optimized topological architecture. The on-chip device is designed on a silicon-on-insulator platform featuring a 220 nm-thick top silicon layer, a 1.5 µm-thick SiO_2_ cladding layer, and a buried SiO_2_ substrate. The compact device consists of two inverse-design mode (de)MUXs and a photonic crystal polarizer. As shown in Ref ^1^, the mode (de)MUXs occupy an area of 2.4 × 3 μm^2^ and achieve a 0.7 dB insertion loss with -17 dB crosstalk over a 60 nm bandwidth. In Ref ^2^, the polarizer is a TM-pass type, which induces a significant loss for the TE mode over a 7.21 μm waveguide. Consequently, the total length of the PIC is approximately 14.24 μm. Figs. S13(b) and S13(c) show the electric field distributions of this compact silicon device obtained through FDTD simulations at a wavelength of 1.55 µm. As illustrated, when the TE₀ mode is input from the left side, the output is the TE₁ mode; conversely, when the TE₀ mode is input from the right side, the output remains the TE₀ mode. These distinct outputs demonstrate the asymmetric switching of the device. Furthermore, the transmission spectra shown in Fig. S13(d) reveal that the minimum transformation loss of this on-chip asymmetric mode switching ranges from 1.60 dB to 2 dB over the 1.52 to 1.58 µm wavelength range, primarily attributed to two mode (de)multiplexers along the path, each introducing approximately 0.7 dB loss. Within this wavelength range, the crosstalk from TE₀ input on the left to TE_1_ output is below -20 dB, and from TE_0_ input on the right to TE_0_ output is below -13 dB, indicating excellent crosstalk performance.

Table 1. Asymmetric mode switching based on the integrated chip.

| Ref | Photonic dimension | Transmission loss ^1^ | Mode purity | Device length | Working wavelength |
| --- | --- | --- | --- | --- | --- |
| ^3^ (Sim) | Mode | < 0.2 dB  < 2.73 dB | > 88 % | 140 μm | 1.2 - 1.7 μm |
| ^3^ (Exp) |  |  | > 91 % |  | 1.53 -1.56 μm |
| ^4^ (Sim) | Mode | < 0.6 dB | > 98.8% | 127 μm | 1.75 - 2.03 μm |
| ^4^ (Exp) |  | < 2.5 dB | > 95 % |  | 1.945 - 2.03 μm |
| ^5^ (Sim) | Mode |  | > 88 % | 69 μm | 1.54 - 1.6 μm |
| ^5^ (Exp) |  | < 3.5 dB | > 90 % |  | 1.54 - 1.565 μm |
| ^6^ (Sim) | Mode | < 1.7 dB | > 90% | 57 μm | 1.5 – 1.7 μm |
| ^6^ (Exp) |  | < 2.5 dB | > 92 % |  | 1.56 - 1.58 μm |
| This work (Sim) | Mode | < 2 dB | > 95 % | 14.24 μm | 1.52 – 1.58 μm |

Some of the results are estimated based on the transmission spectra. Sim: Simulation; Exp: Experiment.

¹Transmission loss refers to the loss of maximum transmission efficiency.

It is also worthwhile to compare the proposed EP-encirclement emulation scheme with previous EP-encirclement approaches. Table 1 summarizes the performance of on-chip asymmetric mode switching in terms of minimum transformation loss, mode purity, device length, and operating bandwidth. In schemes that utilize EP encirclement via Hamiltonian hopping, sufficiently long adiabatic waveguides are designed to allow the system parameters to approach infinity. Based on this concept, a 140 μm-long device was developed to achieve ultra-low mode conversion loss over a 500 nm bandwidth while maintaining a mode purity greater than 88%. Subsequently, metamaterial waveguides were introduced to address wavelength-dependent mode mismatch, enabling broadband, low-loss, and high-purity performance in the 2 μm wavelength range. However, both of these approaches rely on relatively long waveguides. To further reduce device size, alternative strategies such as encircling moving EPs and fast evolution techniques have been proposed, shrinking the device length to 69 μm and 57 μm, respectively. In contrast, our topology-optimized design achieves asymmetric mode switching with a compact device length of only 14.25 μm — just one-quarter the length of previous implementations — while still delivering a wide bandwidth, low transformation loss, and high mode purity. These results clearly demonstrate the superiority of the proposed EP-encirclement emulation scheme for realizing compact, on-chip asymmetric switching devices.

# Reference

1. Chang, W. *et al.* Ultra-compact mode (de) multiplexer based on subwavelength asymmetric Y-junction. *Opt. Express* **26**, 8162 (2018).

2. He, Y., Zhang, Y., Zhang, R., Sun, L. & Su, Y. Ultra-compact and broadband silicon polarizer employing a nanohole array structure. *Opt. Lett.* **46**, 194 (2021).

3. Li, A. *et al.* Hamiltonian Hopping for Efficient Chiral Mode Switching in Encircling Exceptional Points. *Phys. Rev. Lett.* **125**, 187403 (2020).

4. Li, K. *et al.* High‐Performance Chiral Mode Switching Devices Using Silicon Metamaterial Waveguides Beyond 1.55 µm. *Laser & Photonics Reviews* 2401344 (2024) doi:10.1002/lpor.202401344.

5. Liu, Q. *et al.* Efficient Mode Transfer on a Compact Silicon Chip by Encircling Moving Exceptional Points. *Phys. Rev. Lett.* **124**, 153903 (2020).

6. Shu, X. *et al.* Fast encirclement of an exceptional point for highly efficient and compact chiral mode converters. *Nat Commun* **13**, 2123 (2022).
